# Activation of Nuclear Receptor CAR: A Pathway to Delay Aging through Enhanced Capacity for Xenobiotic Resistance

**DOI:** 10.1002/advs.202416823

**Published:** 2025-01-31

**Authors:** Jing Yu, Xiaoyan Gao, Hang Shi, Lijun Zhang, Wenlong Nie, Ruochen Zhang, Minglv Fang, Ying Liu, Yingxuan Yan, Bingbing Fan, Chengyuan Wu, Cheng Huang, Shengjie Fan

**Affiliations:** ^1^ School of Pharmacy Shanghai University of Traditional Chinese Medicine Shanghai 201203 China

**Keywords:** anti‐aging, age‐related neurodegeneration, constitutive androstane receptor (CAR), environmental stimulus, xenobiotic resistance

## Abstract

Environmental factors are linked to aging and age‐related diseases. Emerging evidence suggests that enhancing body's resistance to xenobiotics might be an anti‐aging strategy. The constitutive androstane receptor (CAR) regulates drug‐metabolizing enzymes and transporters, coordinating metabolism and immune responses to adapt to stress triggered by exogenous exposure. However, the impact of activating CAR on aging remains unknown. In this study, *Caenorhabditis elegans* (*C. elegans*), drug‐induced premature aging mice, and senescence accelerated P8 (SAMP8) mice are used as models to explore the effects of CAR activation on lifespan and healthspan, along with the underlying mechanisms. The results showed that hCAR agonist CITCO and mCAR agonist TCPOBOP prolonged the lifespan and healthspan in model organism. The longevity effects of CITCO and TCPOBOP were attenuated in CAR homozygous nhr‐8/daf‐12 mutant *C. elegans* as well as CAR^−/−^ mice. In *C. elegans*, CITCO activated both anti‐stress and detoxification genes, and increased the resistance to environmental adversities. Additionally, the lifespan‐extending and xenobiotic resistant effects of CITCO might be related to the regulation of age‐related pathways. Furthermore, CITCO improved age‐related neurodegeneration in *C. elegans* models. Taken together, the results suggest that the longevity effects of CAR agonists may be related to the enhancement of xenobiotic resistance of animals.

## Graphical abstract

The authors uncovered that targeting the constitutive androstane receptor (CAR) might decelerate the aging process. Employing models like *C. elegans* and mice, they determined that CAR agonists CITCO and TCPOBOP can extend both the lifespan and healthspan. In addition, these agonists fortified the resistance to xenobiotics and mitigated age‐related neurodegeneration, suggesting that activation of CAR could be a potential longevity strategy.

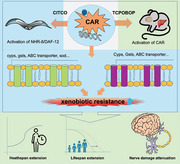

## 1 Introduction

Aging is marked by a gradual deterioration of biological functions as time progresses, leading to a decreased resistance against diverse forms of stress and an increased vulnerability to many diseases.^[^ ^1^ ^]^ Aging and age‐related disorders have become a notable international concern, posing considerable socio‐economic and medical challenges.^[^ ^2^ ^]^ Contemporary studies have shown that biological aging is caused by a combination of genetic and environmental elements.^[^ ^3^ ^]^ The contribution of genes to the lifespan of an individual was estimated to be about 25%, thus non‐genetic and environmental factors might have more than 75% influence in determining the lifespan.^[^ ^4^ ^]^ A variety of radiations in the environment, industry pollution, pesticides, heavy metals, drugs, addictive substances, unhealthy dietary habits, sedentary lifestyles, and psychosocial stresses can accelerate the onset of aging.^[^ ^5^ ^]^ Current studies have suggested that strengthening the body's resistance to unfavorable environmental factors in including heat and toxic substances, can reduce the aging process.^[^ ^3^, ^6^, ^7^ ^]^ For example, long‐lived Ames Pygmy mice enhance resistance to external stressors like paraquat.^[^ ^8^ ^]^ The long‐lived *daf‐2* mutant *C. elegans* exhibits increased resistance to heat stress and oxidative stress.^[^ ^9^ ^]^ The *Drosophila melanogaster* mutant strain *Methuselah* is able to resist heat and paraquat stress.^[^ ^10^ ^]^ Several natural extracts have been reported to boost resistance to environmental stimuli and thereby extend lifespan, such as nomilin, cellulose, apple, and blueberry extracts.^[^ ^11^, ^12^, ^13^, ^14^ ^]^ These data indicate that enhancing the resistance to xenobiotic stress could potentially be an anti‐aging strategy.

Xenobiotic nuclear receptors (NRs) are the sensors by which the organism perceives external stimuli. When the organisms receive stimulation from external stressors, NRs regulate the rapid response of the organism.^[^ ^15^ ^]^ A number of studies have revealed that NRs can play a role in prolonging lifespan through genetic and environmental interference.^[^ ^15^ ^]^ Recent study has shown that Pregnane X receptor (PXR) is capable of enhancing the body's resistance to toxic stimuli in the environment, thereby delaying aging.^[^ ^14^ ^]^ In *C. elegans*, NHR‐8 and DAF‐12 are homologues of mammalian nuclear receptors, which are indispensable for the longevity of *C. elegans*. NHR‐8 is necessary for the lifespan extension by dietary restriction (DR) in *C. elegans*.^[^ ^16^, ^17^ ^]^ In long‐lived *C. elegans* with germline cell deficiencies, the deletion of DAF‐12 reduces lifespan and health status.^[^ ^18^, ^19^ ^]^ DAF‐12 agonists also extend the lifespan of GLP‐1/DAF‐36 mutants.^[^ ^20^ ^]^ In contrast, gain‐of‐function mutations of DAF‐12 display better health and a longer lifespan.^[^ ^21^ ^]^ These studies indicate that nuclear receptors might be an important domain in anti‐aging research.

Constitutive androstane receptor (CAR), belongs to the Nrli family, participates in a wide range of physiological processes. The functions of PXR and CAR are largely overlapped, including the regulation of drug/xenobiotic and bile acid metabolism through drug metabolizing enzyme and transporter expression modulation,^[^ ^22^, ^23^ ^]^ and participating in physiological and pathological conditions of diseases.^[^ ^24^ ^]^ However, PXR is also involved in cholesterol metabolism, inflammation, and cancer. PXR activation may lead to opposite effects on gluconeogenesis in rodents and humans,^[^ ^24^ ^]^ leading to hepatic lipid accumulation and the fatty liver disease.^[^ ^24^ ^]^ In contrast, CAR improves insulin sensitivity, inhibits gluconeogenesis and lipogenesis, and increases brown adipose tissue energy expenditure.^[^ ^25^ ^]^ Thus, the activation of CAR may avoid some side effect of PXR including the control of metabolism (glycolipid, bile acid, steroid, and energy metabolism) and detoxification, among others.^[^ ^26^ ^]^ Many studies indicate that CAR plays a role in enhancing the body's resistance to unfavorable factors in the environment. As a sensor for the toxic by‐products generated by endogenous metabolites and exogenous chemicals, CAR directly regulates the expression of a series of phase I and phase II xenobiotic metabolic enzymes and multidrug transporters,^[^ ^27^, ^28^ ^]^ such as CYP2B, CYP3A and CYP2C, etc.^[^ ^29^, ^30^ ^]^ This is conducive to the detoxification of various environmental chemicals. Increasing evidence suggests that CAR also has the endogenous function of regulating gluconeogenic enzymes, influencing glycolipid metabolism and the pathogenesis of metabolic diseases. Moreover, CAR participates in physiological stress adaptation responses, hormones, and energy balance through glucose and lipid sensing.^[^ ^31^ ^]^ These data imply that CAR not only regulates the transcription of drug‐metabolizing enzymes and transporters but also coordinates energy metabolism and immune responses to adapt to the stress triggered by xenobiotic exposure. Caloric restriction is a longevity intervention confirmed in multiple animal models. It has been reported that caloric restriction may increase CAR expression and activity, coordinating the adaptive response by reducing energy expenditure.^[^ ^32^ ^]^ In long‐lived mice (Ghrhrlit/lit), CAR regulates the expression of phase I detoxification enzyme genes, such as *CYP450s*, *carboxylesterases*, *SDRs*, as well as phase II detoxification enzyme genes *GSTs*, *UDPs*, and *SULTs*.^[^ ^33^ ^]^ However, whether the activation of CAR could extend lifespan and healthspan is uncertain.

CAR displays significant species selectivity in its ligand binding and activation characteristics.^[^ ^34^ ^]^ The hCAR agonist CITCO^[^ ^35^ ^]^ can selectively bind to hCAR and activate the CAR target genes in human hepatocytes,^[^ ^36^, ^37^ ^]^ and effectively enhance the recruitment of coactivators to hCAR LBD by competing with antagonists such as PK11195^[^ ^38^ ^]^ and metformin.^[^ ^39^ ^]^ Thus, CITCO is commonly used as the selective agonist of human CAR. The hepatic amine drug 1,4‐Bis[2‐(3,5‐dichloropyridyloxy)] benzene (TCPOBOP) is the most effective CYP2B inducer and a specific mCAR agonist.^[^ ^40^ ^]^ Here, we show that CAR agonists can extend lifespan and health in *C. elegans* and mice correlated with the enhancement of xenobiotic resistance.

## 2 Results

### 2.1 hCAR Agonist CITCO Extends Lifespan in *C. elegans*

**Figure 1:**
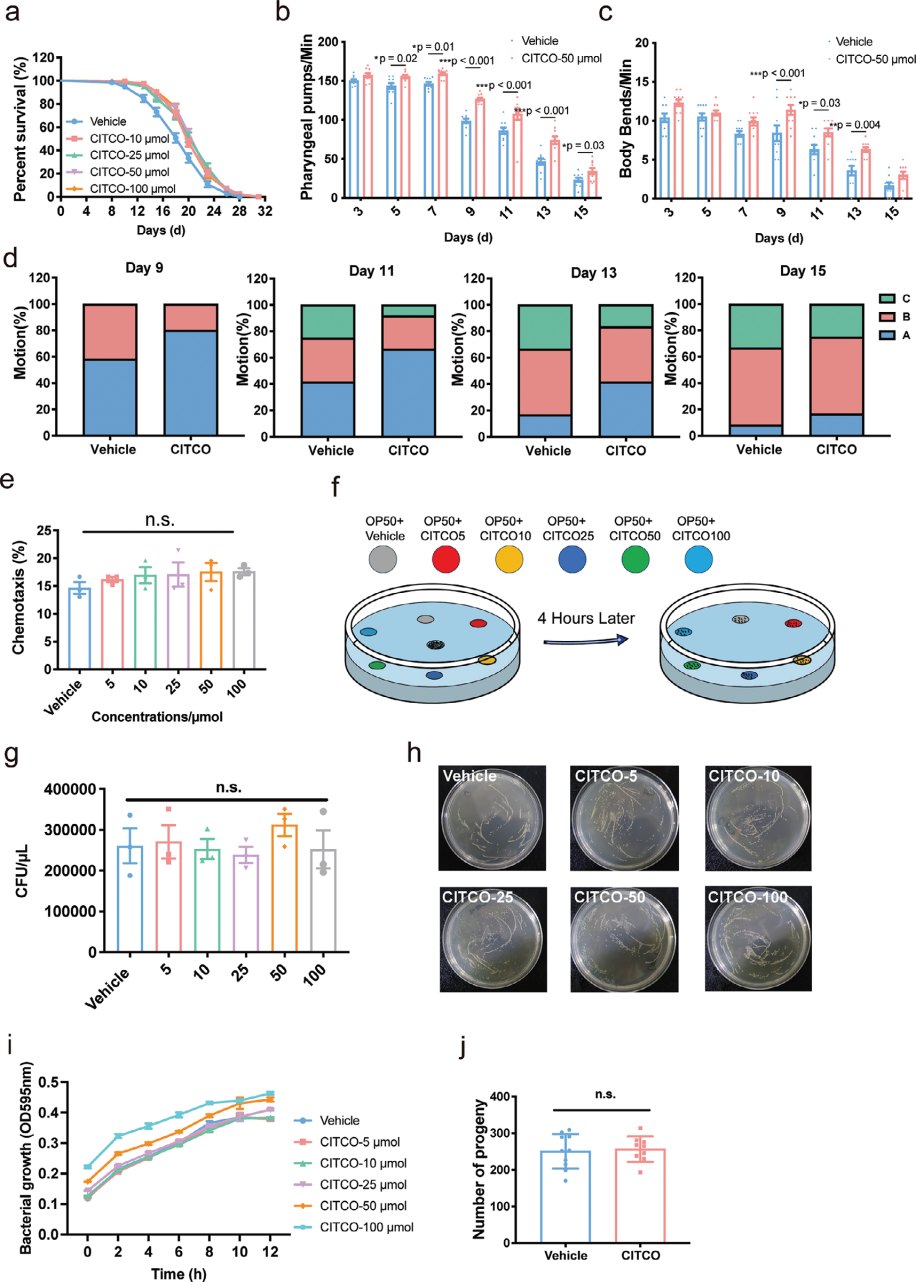
Effect of CITCO on lifespan and healthspan in *C. elegans*. a) Survival curves of *C. elegans* treated with CITCO (log‐rank test, each involving >120 animals). b) The frequency of body bends (*n* = 10 in each group). c) The frequency of the pharyngeal pumps (*n* = 10 in each group). d) Measurement of three levels of motility. Locomotor ability was tested on days 9, 11, 13, and 15 (*n* = 10 in each group). e,f) *C. elegans* “chemotaxis” toward *E. coli OP50* (each involving 50 animals). g,h) Colony‐forming units (CFU) after plating bacteria on plates containing the drug. i) The growth of *E. coli OP50*. j) *C. elegans* reproduction (*n* = 10 in each group). All experiments were carried out at 20 °C. See Table S1 (Supporting Information) for detailed statistical analysis of lifespan data. Significance was analyzed by (b, c, g) two‐way ANOVA. All data were presented as mean ± S.E.M. Compared with Vehicle group, *p < 0.05, **p < 0.01, ***p < 0.001.

Studies have shown that enhanced resistance to exogenous stress is closely related to aging extension.^[^ ^41^, ^42^ ^]^ Thus, we assayed whether hCAR agonist CITCO could prolong the lifespan as well as the healthspan in vivo. Wild‐type N2 *C. elegans* were treated with CITCO. The results showed that CITCO prolonged the mean lifespan by 11.32%, 11.29%, 12.76%, and 11.46% at 10, 25, 50 and 100 µm, respectively (**Figure** **1**a; Table S1, Supporting Information). The locomotor behaviors of *C. elegans*, such as body bending, head swinging and pharyngeal pumping are deteriorated with aging (Figure 1b,c). While, CITCO significantly improved these locomotor behaviors and enhanced the mobility (Figure 1d). These data suggest that CITCO may delay the aging process and extend lifespan and healthspan in *C. elegans*.

DR is well‐known approaches to extend worm lifespan.^[^ ^43^ ^]^ To exclude the possibility that the lifespan‐extending effect of CITCO was due to a decrease in food intake, the bacterial growth and chemotaxis assay were conducted. The chemotaxis assay revealed that *C. elegans* had no dietary preference for OP50 containing vehicle and CITCO (Figure 1e,f). Additionally, as the standard experimental food for *C. elegans*, OP50 was treated with CITCO and grown at 37 °C. We found that CITCO did not affect the growth of OP50 (Figure 1g–i). These data indicate that the lifespan‐extending effect of CITCO is not relevant to food intake. Although CITCO does not alter bacterial growth or the food preference of worms, its effect on overall food intake needs to be investigated to rule out any potential changes in food consumption.

According to the “disposable soma” theory of aging, a decline in fertility may contribute to lifespan extension in *C. elegans*.^[^ ^44^ ^]^ To investigate whether CITCO affects nematode reproduction, we examined the average number of hatchlings and the number of larval progeny (Figure 1h). The results showed no significant differences between CITCO‐treated and control groups. Thus, CITCO may directly contribute to prolongation of lifespan and healthspan in *C. elegans*.

### 2.2 CITCO Improves Stress Resistance and Toxin Resistance in *C. elegans*

**Figure 2:**
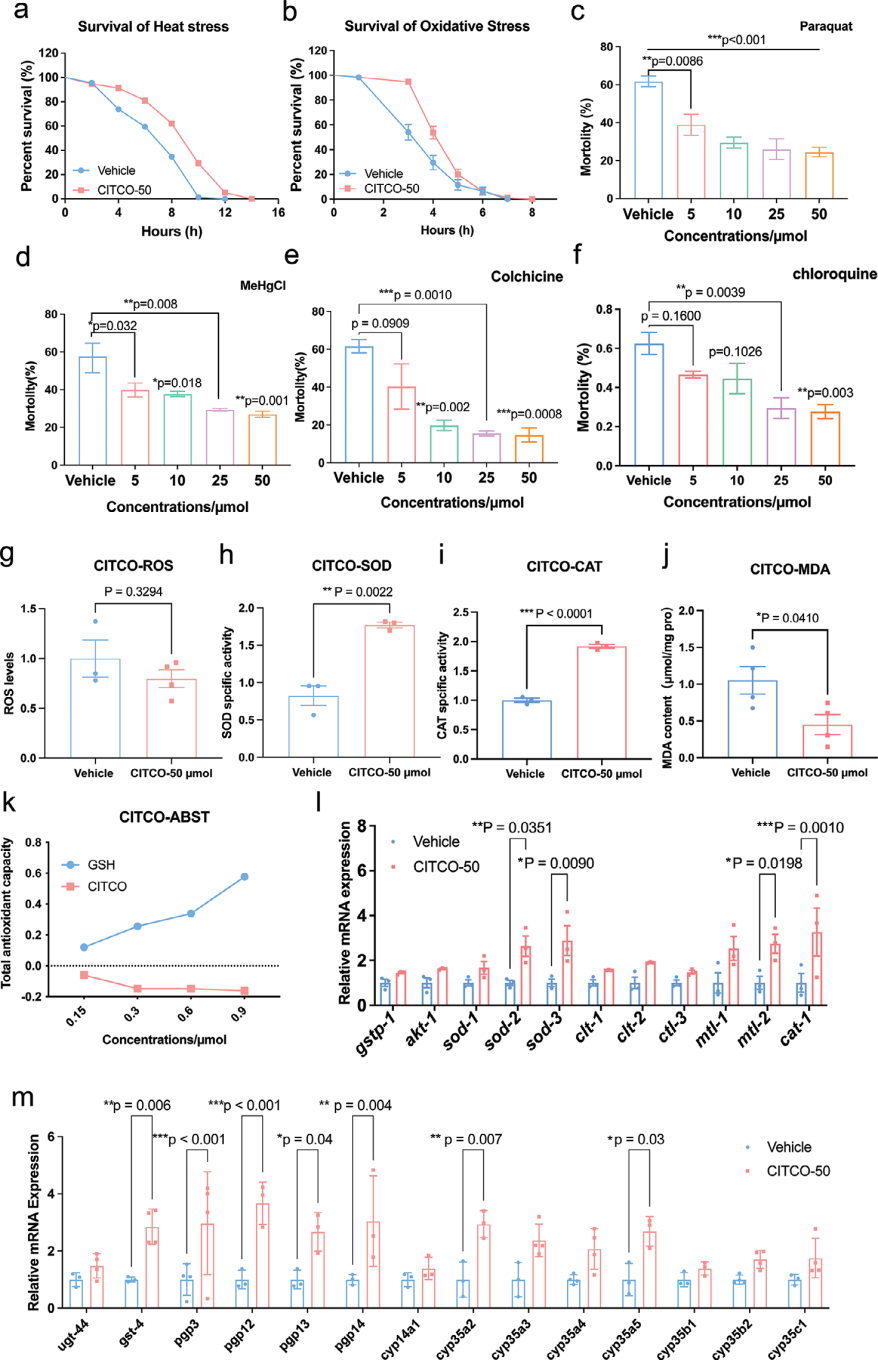
Life‐extending effects of CITCO may be associated with increased xenobiotic stress resistance. a) Survival curves of *C. elegans* under 35 °C for heat stress analysis (*n* = 50–60). The experiment was repeated 3 times. b) Survival curves of N2 *C. elegans* exposed to hydrogen peroxide (final concentration: 8 mm) for oxidative stress analysis (*n* = 60–70 *C. elegans*). The experiment was repeated 3 times. The survival data were analyzed by lo‐rank test, and the detailed data were concluded in Table S2 (Supporting Information). c) The resistance to oxidative stress of N2 *C. elegans* to 200 mm paraquat (PQ). About 50 *C. elegans* in each group. d) The survival rate of *C. elegans* treated by 2 µm MeHgCl. e) The livability of *C. elegans* exposed to 4 mm colchicine (CC). f) The livability of *C. elegans* exposed to 4 mm chloroquine (CQ). g) ROS contents in *C. elegans* (*n* = 3). h) Superoxide dismutase (SOD) activity in *C. elegans*. i) Catalase activity. j) Levels of malondialdehyde (MDA). k) Total antioxidant capacity of CITCO and glutathione (GSH). l) qRT‐PCR analysis of stress resistance‐related gene expression (*n* = 3). m) qRT‐PCR analysis of detoxification gene expression (*n* = 4). Significance was analyzed by Two‐tailed unpaired Student's *t*‐test (d, e), Two‐way ANOVA. Data were expressed as mean ± S.E.M. and *n* ≥ 3 in each experiment. Compared with Vehicle group, *p < 0.05, **p < 0.01, ***p < 0.001.

The increased longevity and healthspan are usually accompanied with enhanced tolerance to environmental insults.^[^ ^42^, ^45^ ^]^ To assay CITCO's stress resistance effect, we observed CITCO‐treated nematodes under heat and oxidative stress conditions. For the heat stress experiment, *C. elegans* were incubated in a 35 °C incubator until death. And the results showed that the survival rates were increased by 24.18% after the treatment of CITCO (**Figure** **2**a, Table S2, Supporting Information). For the oxidative stress experiment, we exposed *C. elegans* to NGM plates containing 8 mm hydrogen peroxide (H_2_O_2_) and placed them in a 20 °C incubator until death. The result showed a 24.87% increase in survival rate after CITCO treatment (Figure 2b, Table S2, Supporting Information). The environmental toxins such as paraquat (PQ), methylmercury chloride (MeHgCl), colchicine (CC), and chloroquine (CQ) are involved in the aging process and the pathogenesis of senile diseases. While, CITCO treatment was able to dose‐dependently increase the survival rate of animals exposed to PQ, MeHgCl, CC and CQ (Figure 2c–f), suggesting that CITCO can protect *C. elegans* from a wide range of chemical toxic stresses.

Increased stress resistance can protect the body from a variety of harmful stimuli. To further investigate the relationship between the lifespan‐extending effect of CITCO and the anti‐stress effect, we examined the effect of CITCO on the stress resistance of *C. elegans*. Excessive ROS levels contribute to aging.^[^ ^46^ ^]^ In aged *C. elegans*, the CITCO‐treated group showed a non‐significant trend (*p* = 0.329) toward reduced ROS levels compared to the control group (Figure 2g). We also examined the content of CAT, MDA and SOD, the indicators of antioxidant capacity (Figure 2g–j). The results showed that CITCO significantly reduced MDA levels (Figure 2j) and increased CAT and SOD enzyme levels (Figure 2h,i). Next, we tested the antioxidant capacity of CITCO using ABTS cation scavenging assay, which showed opposite results to that of the positive control glutathione (GSH) (Figure 2k), suggesting that CITCO has no antioxidant capacity. In addition, CITCO treatment elevated the mRNA levels of several stress resistance‐related genes in *C. elegans*, including *sod‐2*, *sod‐s*, *mtl‐3*, and *cat‐1* (Figure 2l). These data suggest that the role of CITCO in exerting antioxidant capacity may be related to its involvement in a complex metabolic regulatory network in *C. elegans*, such as the promotion of antioxidant production or enhancement of antioxidant enzyme activity.

The endogenous detoxification system includes Phase I/II detoxification enzymes and Phase III ABC transporters.^[^ ^47^, ^48^ ^]^ To understand CITCO's toxic resistance effect, mRNA levels of detoxification genes were tested with qRT‐PCR analysis. The result showed that Phase I enzymes *cyp35a2* and *cyp35a5*, Phase II *gst‐4* and Phase III *pgp‐3, pgp‐12, pgp‐13*, and *pgp‐14* were upregulated in *C. elegans* after CITCO treatment (Figure 2m). Overall, these results suggest that the positive regulation of CITCO on detoxification gene expression for stress resistance may be one of the key mechanisms for its anti‐aging effect.

### 2.3 The Anti‐Aging Effects of CITCO Depend on NHR‐8/DAF‐12

**Figure 3:**
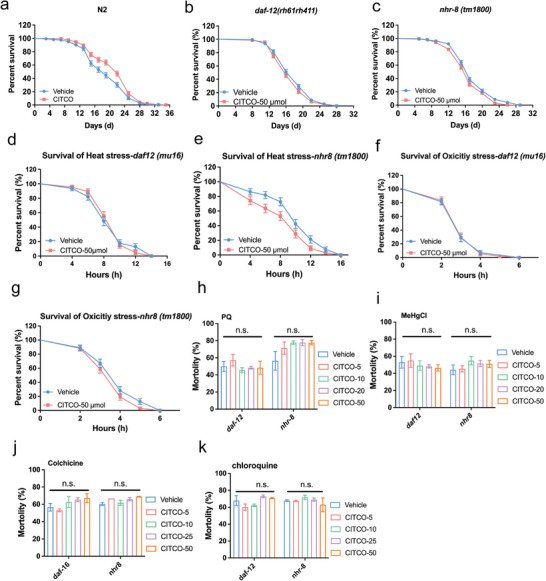
CITCO exerts anti‐aging and anti‐xenobiotic stimulatory impacts in the worm through nuclear hormone receptors NHR‐8/DAF‐12. a) The survival curves of N2 *C. elegans*, b) The survival curves of *daf‐12 (rh61rh411)*. c) The survival curves of *nhr‐8 (tm1800)*. All lifespans were performed at 20 °C and analyzed by log‐rank, *n* = 162–269. The detailed lifespan data were expressed in Table S3 (Supporting Information). d) Survival curves of *daf‐12 (rh61rh411)* under 35 °C for heat stress analysis (*n* = 60–70 *C. elegans*). e) Survival curves of *nhr‐8 (tm1800)* under 35 °C for heat stress analysis (*n* = 60–70 *C. elegans*). f) Survival curves of *daf‐12 (rh61rh411)* exposed to hydrogen peroxide (final concentration: 8 mm) for oxidative stress analysis (*n* = 50–70 *C. elegans*). g) Survival curves of *nhr‐8 (tm1800)* exposed to hydrogen peroxide (*n* = 60–70 *C. elegans*). The survival data were analyzed by log‐rank test, and the detailed data were concluded in Table S4 (Supporting Information). h) The toxic resistance of *daf‐12 (rh61rh411)* and *nhr‐8 (tm1800)* mutants to 200 mm paraquat (PQ). About 50 *C. elegans* in each dose of CITCO. i) The survival rate of *daf‐12 (rh61rh411)* and *nhr‐8 (tm1800) C. elegans* treated by 2 µm MeHgCl. j) The livability of *daf‐12 (rh61rh411)* and *nhr‐8 (tm1800)* mutant exposed to 4 mm colchicine (CC). k) The livability of *daf‐12 (rh61rh411)* and *nhr‐8 (tm1800)* mutants exposed to 4 mm chloroquine (CQ). Significance was analyzed by One‐way ANOVA (h, i, j, k). Data were expressed as mean ± S.E.M. and *n* ≥ 3 in each experiment. Compared with Vehicle group, *p < 0.05, **p < 0.01, ***p < 0.001.

In *C. elegans*, NHRs DAF‐12, NHR‐8, and NHR‐48 are structurally and functionally homologous to mammalian PXR/CAR.^[^ ^22^, ^49^ ^]^ It has been reported that NHR‐8 and DAF‐12 play important roles in lifespan extension and resistance to exogenous toxic stimuli.^[^ ^50^ ^]^ To investigate whether NHRs signaling is required for the lifespan prolonging effects of CITCO, the role of DAF‐12 and NHR‐8 were tested during CITCO treatment. Interestingly, unlike in N2 *C. elegans* (**Figure** **3**a, Table S3, Supporting Information), CITCO treatment did not result in an increased in lifespan in *daf‐12 (rh61rh411)* mutant compared to vehicle treatment (Figure 3b, Table S3, Supporting Information). Similar results were observed in *nhr‐8 (tm1800)* mutants (Figure 3c, Table S3, Supporting Information). These data indicate that the longer lifespan after CITCO treatment may rely on NHR‐8 and DAF‐12 signaling.

To explore whether the xenobiotic resistance impacts of CITCO depend on NHR‐8 and DAF‐12, we performed heat stress and oxidative stress experiments on *nhr‐8 (tm1800)* and *daf‐12 (rh61rh411)* mutant *C. elegans*, and the results showed that CITCO failed to extend the survival rates (Figure 3d–g, Table S4, Supporting Information). Then, we exposed N2 *C. elegans* to PQ, MeHgCl, CC, and CQ, the rates of death of *daf‐12* and *nhr‐8* mutant *C. elegans* were not increased (Figure 3h–k). These data indicate that lifespan‐extending effects of CITCO may be linked to NHR‐8 and DAF‐12‐mediated stress resistance.

### 2.4 CITCO Interacts with Multiple Aging‐Related Signaling Pathways

**Figure 4:**
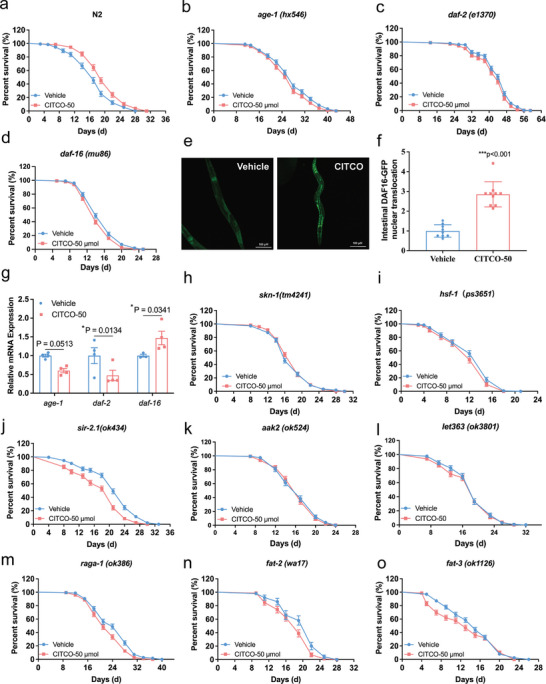
Multiple aging‐related signaling pathways modulated by CITCO. a) Survival curves of N2 *C. elegans*. b) The survival curve of *age‐1 (hx546)*, c) The survival curve of *daf‐2 (e1370)*. d) The survival curve of *daf‐16 (mu86)*. e,f) The nuclear translocalization of DAF‐16:: GFP (*muIs109*) *C. elegans*. At least 10 *C. elegans* were analyzed. g) The expression of the genes *daf‐16*, *age‐1*, and *daf‐2* tested by qRT‐PCR. h) The survival curve of *skn‐1 (tm4241)*. i) The survival curve of *hsf‐1 (ps3651)*. j) The survival curve of *sir‐2.1 (ok434)*. k) The survival curve of *aak‐2 (ok524)*. l) The survival curve of *let‐363 (ok3018)*. m) The survival curve of *raga‐1 (ok386)*. n) The survival curve of *fat‐2 (wa17)*. o) The survival curve of *fat‐3 (ok1126) n* = 107–215 *C. elegans* for each group. Lifespan assays were performed at 20 °C except *fat‐2*, which was maintained at 25 °C, and all lifespan assays were analyzed by log‐rank test. Data were expressed as mean lifespan ± S.E.M. The detailed lifespan results were listed in Table S5 (Supporting Information). Significance was analyzed by One‐way ANOVA (f). The data were analyzed by Two‐tailed unpaired Student's *t*‐test (g). Compared with Vehicle group, *p < 0.05, ***p < 0.001.

Insulin/insulin‐like growth factor signaling (IIS) pathway is an evolutionarily conserved nutrient‐sensing network that plays an important role in nematode tolerance to various stresses and longevity.^[^ ^41^ ^]^ It has been discovered that CAR inhibits the insulin‐dependent activity of FOXO1 in mammals.^[^ ^51^ ^]^ Moreover, in non‐mammalian animals, the nuclear receptor contributes to enhanced exogenous resistance and longevity in IIS mutants.^[^ ^52^, ^53^ ^]^ Thus, we assayed the lifespan of key regulators in the IIS pathway, *daf‐2*, *age‐1* (mammalian PI3K homolog) and *daf‐16* (mammalian FOXO) mutants. Our findings revealed that CITCO did not extend the lifespan of *daf‐16 (mu86)*, *daf‐2 (e1370)*, and *age‐1 (hx546)* mutant (**Figure** **4**a–d, Table S5, Supporting Information). Furthermore, CITCO promoted DAF‐16::GFP nuclear translocation (Figure 4e,f), upregulated the mRNA levels of *daf‐16*, and downregulated the expression of the *daf‐2* (Figure 4g). Contemporary studies have indicated that *hsf‐1* and *skn‐1* are pivotal genes that boost the IIS signaling pathway to govern xenoresistance and are downstream targets of *daf‐16* in *C. elegans*.^[^ ^54^, ^55^ ^]^ Interestingly, the lifespan extension effect of CITCO in *skn‐1 (tm4241*) and *hsf‐1 (ps3651) C. elegans* were fully blocked (Figure 4h,i, Table S5, Supporting Information). In *C. elegans*, *sir‐2.1* homologous to human SIRT1, can regulate the activity of daf‐16/FOXO directly or indirectly through deacetylation, controlling the cellular response to stress.^[^ ^3^, ^56^ ^]^ When *sir‐2.1 (ok434) C. elegans* were exposed to CITCO, the average lifespan was decreased compared to the vehicle group (Figure 4j, Table S5, Supporting Information).

Many studies have shown that activation of AMPK, a key regulator of cellular energy metabolism, can prolong lifespan and increase anti‐stress ability.^[^ ^57^, ^58^ ^]^ The specific and direct interaction mechanism between CAR and AMPK remains undefined. Nevertheless, CAR is assumed an important role in coordinating energy metabolism, particularly dietary restriction,^[^ ^59^, ^60^ ^]^ which might act synergistically with AMPK to regulate energy metabolism and maintain cellular and organismal energy homeostasis. To analyze whether CAR is associated with AMPK signaling, we conducted lifespan experiments on *aak‐2 (ok524)*, the AMPK homologous gene mutant *C. elegans*. The results showed that compared to those in the vehicle group, the lifespan‐extending effects of CITCO were inhibited (Figure 4k, Table S5, Supporting Information). In *C. elegans*, mTOR signaling pathway is regulated by NHR‐8/DAF12 as well as interacting with the AMPK signaling pathway.^[^ ^17^, ^61^ ^]^ Recent research has demonstrated that the activation of CAR is indispensable for adapting to nutritional stresses.^[^ ^32^ ^]^ CITCO did not extend the lifespan in *C. elegans let363*, the homolog of TOR (Figure 4l), and *raga‐1*, mTORC1 signaling component RagA/B homologue (Figure 4m, Table S5, Supporting Information). Additionally, the long‐lived *eat‐2 (ad1116)* mutant, which exhibits a decreased pharyngeal pumping rate for reduced food intake and is utilized as a DR model, did not show an increase in survival rates following CITCO treatment (Figure S2 and Table S5, Supporting Information).

Mitochondrial morphology and energy metabolism are suggested to have an interdependent relationship, and regulating the integrity of mitochondrial function can help the organism adapt to environmental stresses.^[^ ^62^ ^]^ To further explore the effects of CITCO on energy metabolism in *C. elegans*, *foxSi16 [myo‐3p::tomm‐20::mKate2::HA::tbb‐2 3′ UTR]* strain was involved, which muscular mitochondria are labeled with a reporter. Compared with vehicle group, CITCO increased mitochondrial content in elder *C. elegans* at the age of Day 7 and Day 14 (Figure S1a,b, Supporting Information), indicating the integrity of mitochondria, a biomarker of senescence in *C. elegans*, was protected under CITCO supplementation. These data indicate that energy metabolism might be involved in CITCO extending lifespan.

It has been reported that lipid signaling molecules can act as health regulators in *C. elegans* and can be modulated by nhr‐8 and daf‐12 in *C. elegans*.^[^ ^15^ ^]^ CAR participates in the regulation of glycolipid metabolism and exerts a regulatory effect on triglyceride levels.^[^ ^63^ ^]^ To test whether the life‐extending effect of CITCO is related to lipid metabolism, we performed lifespan assays on nematodes deficient in the desaturases FAT‐2 (Δ12 unsaturase) and FAT‐3 (Δ6 unsaturase), including *fat‐2 (wa17)*, *fat‐3 (ok1126)*. The longevity effects of CITCO were markedly attenuated (Figure 4n,o, Table S5, Supporting Information). Taken together, these data suggests that multiple aging‐related signaling might be involved in CITCO extending lifespan, which is related to the increase of stress resistance of nematodes.

### 2.5 The Effects of CITCO on Aging‐Related Neurodegeneration

**Figure 5:**
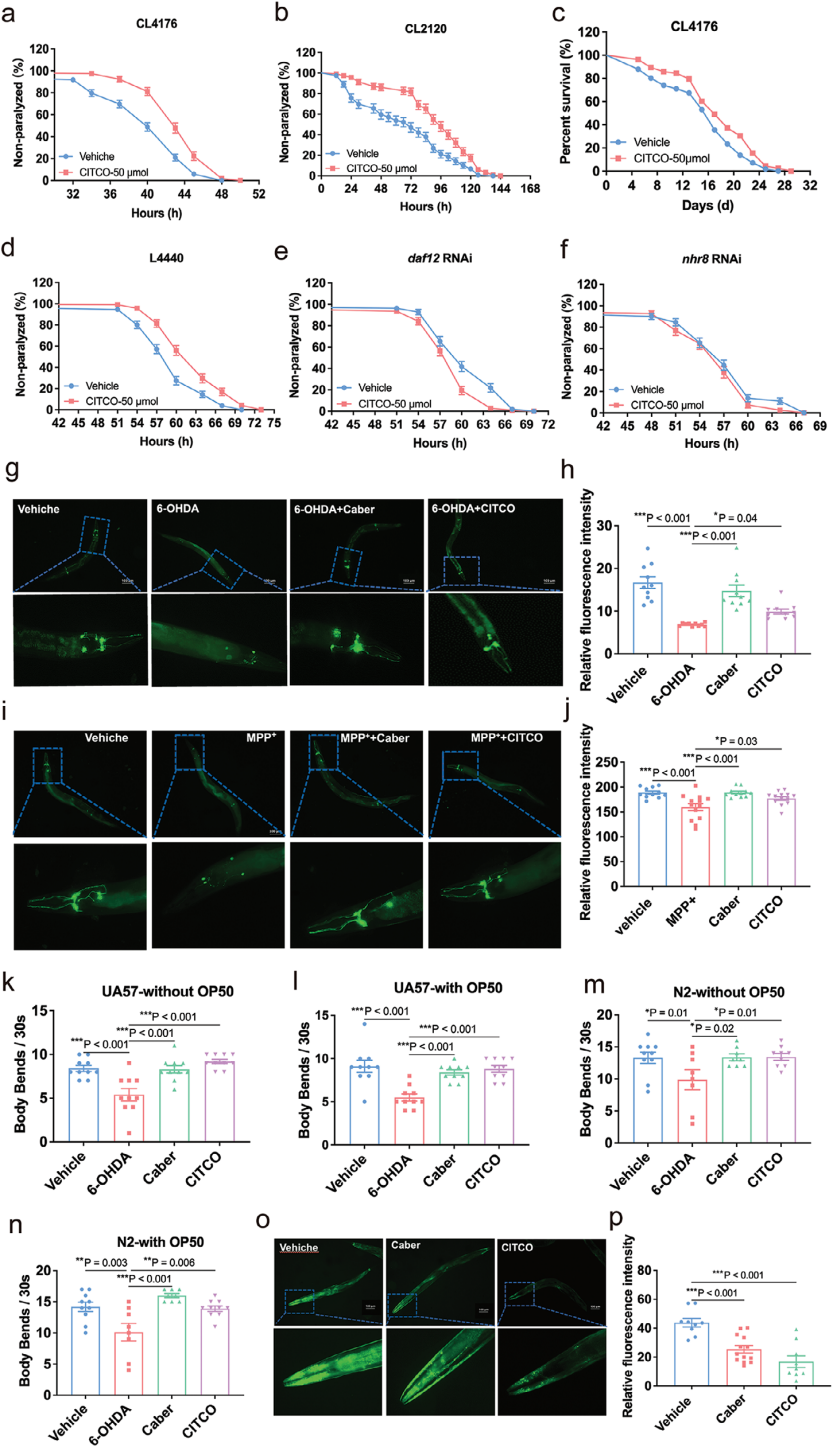
Effects of CITCO on aging‐related neurodegeneration. a) Paralysis of CL4176 *C. elegans*. b) Paralysis of CL2120 *C. elegans*. c) Survival curves of CL4176 *C. elegans*. Lifespan assays were performed at 25 °C. d–f) Paralysis of CL4176 fed with L4440, *daf12* RNAi and *nhr8* RNAi. g–j) CITCO ameliorates 6‐OHDA and MPP^+^‐induced Dopamine neuron degeneration in UA57 *C. elegans*. k,l) CITCO ameliorates 6‐OHDA‐induced UA57 *C. elegans* locomotion deficits (*n* = 10). m,n) Locomotion of N2 *C. elegans* (*n* = 10). o,p) The accumulation of α‐synuclein in the muscle tissue of the body wall of NL5901 *C. elegans* (*n* = 10). Positive control groups were treated with cabergoline (Caber). All lifespan and paralysis assays were analyzed by log‐rank test, *n* = 82–172 *C. elegans* for each group. Data were expressed as mean lifespan ± S.E.M. The detailed lifespan and paralysis results were listed in Tables S6 and S7 (Supporting Information). Significance was analyzed by One‐way ANOVA (f). The data were analyzed by Two‐tailed unpaired Student's *t*‐test. Compared with Vehicle group in (a–f, p). compared with 6‐OHDA group in (h, k–n), compared with MPP^+^ group in (j) *p < 0.05, **p < 0.01, ***p < 0.001.

Considering the promising anti‐aging activity of CITCO, we further explored its role in Parkinson's disease (PD) and Alzheimer's disease (AD) models of *C. elegans*. In AD brain, one of the most obvious pathologic features is amyloid (Aβ) deposition.^[^ ^64^ ^]^ We found that in the CL4176 and CL2120 strains that expression human Aβ in muscle cells, CITCO treatment significantly prolonged the time to the onset of paralysis compared to the control group (**Figure** **5**a,b, Table S6, Supporting Information). Positive correlation was found between lifespan extension and protection against Aβ toxicity,^[^ ^65^, ^66^ ^]^ thus we investigated the effect of CITCO on lifespan extension by performing survival assay using CL4176. Interestingly, CITCO treatment significantly prolonged the lifespan under Aβ toxicity (Figure 5c, Table S7, Supporting Information). Hence, CITCO may reduce Aβ toxicity in *C. elegans*.

To further investigate whether the decrease of Aβ toxic effects by CITCO is associated with *nhr‐8* and *daf‐12*, the gene *daf‐12* and *nhr‐8* were knocked down using RNAi in CL4176 *C. elegans* and treated with CITCO. The results showed that the effects of CITCO on paralysis were attenuated in *daf‐12* or *nhr‐8* deficient *C. elegans* (Figure 5e,f, Table S6, Supporting Information) when compared to those of *C. elegans* treated with an control vector (L4440) (Figure 5d, Table S6, Supporting Information). Collectively, these findings suggest that *daf‐12* and *nhr‐8* are required for the effect of CITCO in reducing A*β* toxicity.

Age‐related loss of dopamine neurons is an important feature of PD. UA57 (*pkls2386*) is a classic strain for PD studies marked dopamine neurons with GFP. Here, we treated UA57 *C. elegans* with 6‐OHDA and MPP^+^ to induce the degeneration of dopamine neurons.^[^ ^67^ ^]^ The results showed that the fluorescence intensity was increased in the CITCO‐treated group compared to that in the models (Figure 5g–j). Additionally, by observing the body bends of nematodes, it was found that CITCO improved the motor incoordination of N2 and UA57 caused by 6‐OHDA (Figure 7k–n). It suggests that CITCO can prevent the damage of dopamine neurons caused by the neurotoxic substance, and play a neuroprotective role in pathological conditions. Pathological α‐synuclein aggregation has been reported to be associated with the decline of dopamine neurons.^[^ ^68^ ^]^ To further confirm the effects of CITCO on PD, we assayed the effects of CITCO on nematode NL5901 (*bals4*), a transgenic model of PD created by inserting human *α‐synuclein* gene with YFP into *unc‐54* promoter.^[^ ^67^ ^]^ It was found that the CITCO‐treatment attenuated pathological α‐synuclein aggregation compared to the control group (Figure 7o,p). Together, CITCO may have neuroprotective effects and can prolong the lifespan of *C. elegans* in age‐related neurodegenerative conditions.

### 2.6 The mCAR Agonist TCPOBOP Improves Healthspan of D‐Galactose Induced Early‐Senescence Mice

**Figure 6:**
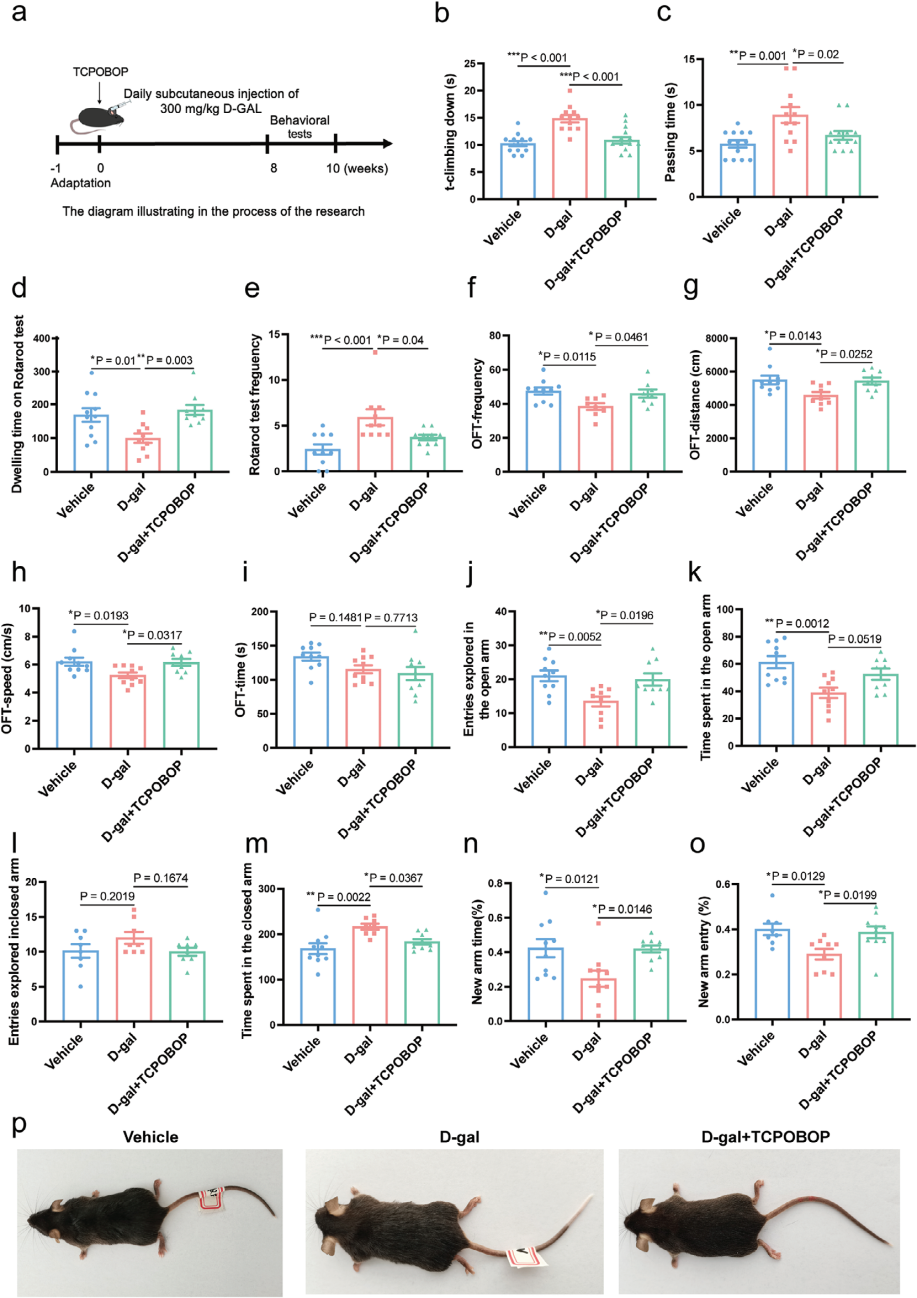
TCPOBOP improves healthspan in D‐galactose induced accelerated aging mice. a) The diagram illustration of the experimental process (*n* = 10 in each group). b) T‐climbing down in the pole test. c) The turning time on the ball in the pole test. d) The time of the first fall of the rotarod test. e) Fall frequencies of the rotarod test. f) Number of entries travelled in the center of the open field test. g) Total distance traveled in the open filed test. h) Speed in the center of the open field test. i) Duration in the center of the open field test. j) Entries explored in the open arm of the elevated plus maze. k) Time spent in the open arm of the elevated plus maze. l) Entries explored in the closed arm of the elevated plus maze. m) Time spent in the closed arm of the elevated plus maze. n) Time spent in the new arm of the Y maze test. o) Number of entries in the new arm of the Y maze test. p) The images of mice. Data were expressed as mean ± S.E.M and analyzed by one‐way ANOVA (*n* = 8–10 in each group). Compared with D‐gal group, *p < 0.05, **p < 0.01, ***p < 0.001. D‐gal, D‐galactose.

The subacute senescence model induced by D‐galactose in rodents was first reported by Xu,^[^ ^69^ ^]^ who found that mice constantly injected with D‐galactose exhibited the signatures of aging. Chronic administration of D‐galactose induces accelerated senescence and may be related to the increased inflammation and oxidative stress via accumulation of ROS, leading to cell injury and aging aspects in multiple organs.^[^ ^70^, ^71^ ^]^ To test whether TCPOBOP could improve healthspan in toxin‐induced senescence, we used D‐galactose to mimic the symptoms of human aging in mice. As we know, impaired cognition, increased anxiety and depression‐like behaviors and decreased motor activity are common age‐induced behavioral changes.^[^ ^72^, ^73^, ^74^ ^]^ In the pole test and beam balance test, D‐galactose‐treated mice spent more time on climbing down from the pole and passing time than control mice. Following TCCPOBOP treatment, the times of T‐climbing and passing time were shortened (**Figure** **6**b,c). The rotarod test revealed that D‐galactose‐treated mice significantly increased the first time, and the total frequencies fell from the rotarod compared with control mice, which were all reversed by TCPOBOP treatment (Figure 6d,e). These data indicate that TCPOBOP could improve D‐galactose‐induced movement disorders. In addition, to investigate whether TCPOBOP treatment could alleviate anxiety, the open field test (OFT) and elevated plus maze test were performed. In the OFT, D‐galactose‐induced mice had short traveled total distance and frequencies in the center. TCPOBOP significantly enhanced total travelled distance and entries in the center (Figure 6f,g). Moreover, D‐galactose‐treated mice showed a slowing of locomotion in the central region, which was significantly ameliorated by TCPOBOP intervention (Figure 6h,i), in line with the previously demonstrated ability of TCPOBOP to improve locomotion in D‐galactose‐treated mice (Figure 6b–e). Similarly, elevated plus maze test showed that D‐galactose‐treated mice displayed significant anxiety‐like behaviors, as indicated by a decrease in the percentage of time spent, the number of entries into the open arms compared to control mice, and an increase in the percentage of time spent, the number of entries into the closed arms (Figure 6j–m). In contrast, TCPOBOP intervention decreased the anxiety‐like behaviors of D‐galactose‐treated mice (Figure 6j–m). The Y‐maze test was used to measure working memory and exploration activity.^[^ ^75^ ^]^ D‐galactose reduced the exploratory time and frequencies in the new arm, while TCPOBOP significantly increased the exploratory time in the new arm (Figure 6n,o).

**Figure 7:**
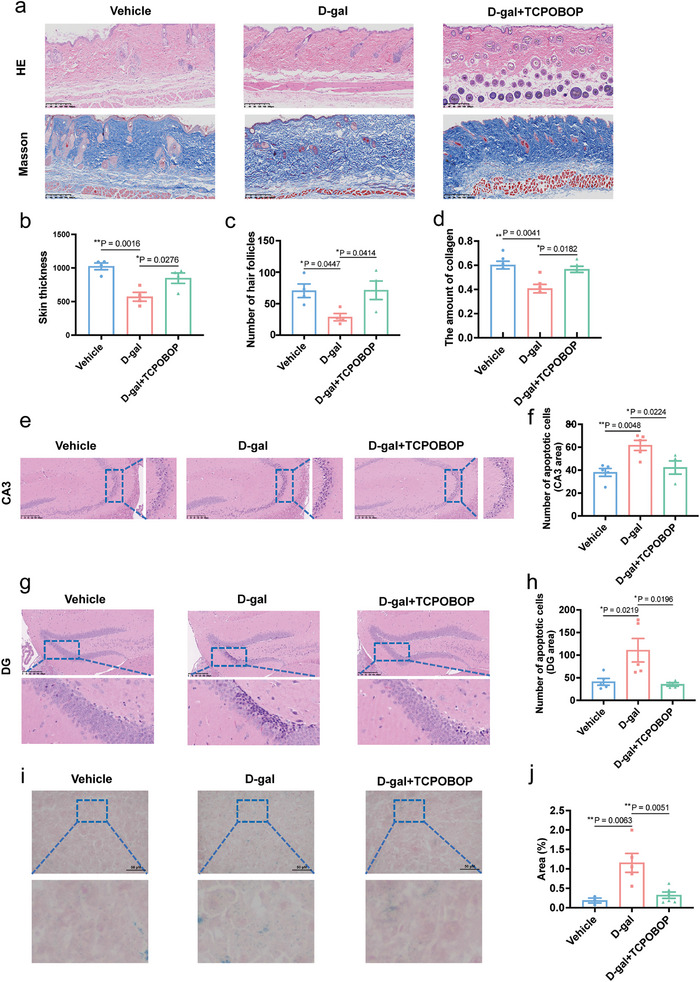
TCPOBOP improves healthspan in D‐galactose induced accelerated aging mice. a) H&E staining of the skin. b) Masson staining and quantitative analysis of the skin. c) The quantitative analysis of skin thickness. d) The quantitative analysis of the number of the hair follicles. e) H&E staining of the CA3 area of the hippocampus. f) The quantification of damaged neurons in the CA3 area. g) H&E staining of the CA3 area of the hippocampus. h) The quantification of damaged neurons in the CA3 area. i) SA‐β‐gal staining of mouse liver tissue. j) The area of positive SA‐β‐gal staining. Data were expressed as mean ± S.E.M and analyzed by one‐way ANOVA (*n* = 3–5 in each group). Compared with D‐gal group, *p < 0.05, **p < 0.01, ***p < 0.001. D‐gal, D‐galactose.

Hair graying and skin aging are indicators of physiological aging symptoms.^[^ ^76^ ^]^ Here, we found that D‐galactose‐treated mice had reduced gray hairs to more hair coloration compared to the control group (Figure 6p). Furthermore, we stained the skin of the mice with Masson and H&E stains. The results demonstrated that compared with the vehicle group, D‐galactose‐treated mice had reduced and sparser distribution of collagen fibers in the skin, poor directionality, a significant reduction in the number of hair follicles, and a significant thinning of the skin thickness (**Figure** **7**a–d). However, TCPOBOP intervention significantly improved the phenomena in the skin (Figures 6p and 7a–d). In conclusion, D‐galactose induced a series of structural changes and functional aging in mouse skin, and TCPOBOP significantly improved skin aging. Learning and memory capacity is correlated with the changes of the hippocampus during ageing.^[^ ^77^ ^]^ To further explore the effects of TCPOBOP on cognitive function, we assessed hippocampal neuronal damage by H&E staining. D‐galactose induced neuronal damage in the CA3 and the dentate gyrus regions, while TCPOBOP reduced damaged neurons in the CA3 and the dentate gyrus regions of the hippocampus (Figure 7e–h). One of the most widely used biomarkers of senescence is senescence‐associated β‐galactosidase (SA‐β‐gal).^[^ ^78^ ^]^ Liver senescence is inextricably linked to the whole organism's response to aging.^[^ ^79^ ^]^ Thus, we stained the livers of mice for SA‐β‐Gal, and found that D‐galactose‐treated mouse liver tissue increased the area of positive staining, while the area of positive staining was decreased after TCPOBOP intervention (Figure 7i,j). These data suggest that TCPOBOP treatment can attenuate D‐galactose‐induced hepatic cellular senescence.

### 2.7 TCPOBOP Enhances Detoxification in D‐Galactose‐Treated Mice

**Figure 8:**
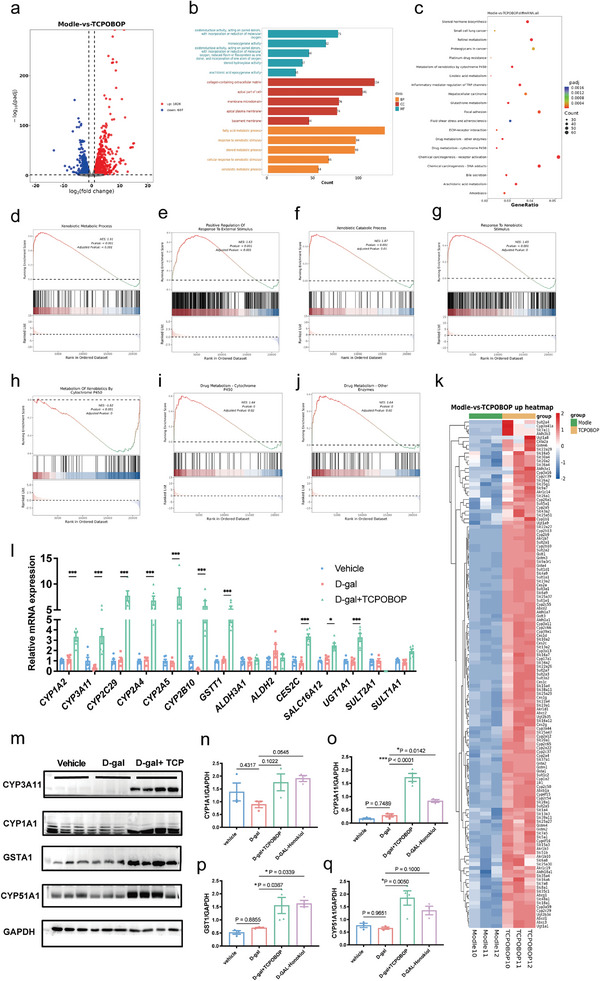
TCPOBOP activates detoxification machinery in D‐galactose induced accelerated aging mice. a) Volcano plot analysis of the samples. b) Gene ontology (GO) terms that were enriched in control and TCPOBOP group mice, including biological process, Cellular component, and Molecular function. c) The Kyoto Encyclopedia of Genes and Genomes (KEGG) analysis of enriched pathways in TCPOBOP‐treated mice. d–j) Functional gene set enrichment analysis (GSEA) of xenobiotic metabolic process, positive regulation of response to external stimulus, xenobiotic catabolic process, response to xenobiotic stimulus, metabolism of xenobiotics by cytochrome P450, drug metabolism‐cytochrome P450, drug metabolism‐other enzymes (*n* = 3). k) The RNA‐seq hierarchical clustering heatmap showing differentially expressed genes from D‐galactose‐treated and TCPOBOP‐treated mouse liver. l) qRT‐PCR analysis of genes enriched in xenobiotics and drug metabolism pathways (*n* = 5). m) Protein levels by western blotting analysis. n–q) The quantification of CYP1A1, CYP3A4, GSTA1, and CYP51A1 proteins, *n* = 3–4 in each group. Significance was analyzed by Two‐tailed unpaired Student's *t*‐test (i), and significance was analyzed by one‐way ANOVA (n–q). Data were presented as means ± S.E.M. Compared with D‐gal group, *p < 0.05, **p < 0.01, ***p < 0.001. D‐gal, D‐galactose.

The most important function of CAR is to serve as a stress sensor for xenobiotics and metabolic stress.^[^ ^80^, ^81^ ^]^ Recently, it has been identified that 17 known lifespan‐extending interventions may induce common transcriptome markers including an increase in the expression of detoxification genes in the liver of mice.^[^ ^82^ ^]^ To investigate the transcriptome in the liver, the mice were treated with TCPOBOP, and the liver tissues were analyzed by RNA‐sequence technique. Volcano plot showed that there were 1826 genes were upregulated, and 607 genes were downregulated following TCPOBOP treatment (**Figure** **8**a). GO terms, including biological process, molecular function, and cellular component, showed that oxidoreductase activity, monooxygenase activity, steroid hydroxylase activity, arachidonic acid epoxygenase activity, fatty acid metabolic process, response to xenobiotic stimulus, steroid metabolic process, cellular response to xenobiotic stimulus and xenobiotic metabolic process were enriched (Figure 8b). KEGG enrichment analysis revealed that after TCPOBOP intervention, mouse livers exhibited significant upregulation of gene expression characteristics commonly found in the livers of long‐lived mice, such as steroid hormone biosynthesis, retinol metabolism, metabolism of cytochrome P450 to xenobiotics, ECM–receptor interactions, apoptosis, age‐related diseases fluid shear stress and atherosclerosis (Figure 8c). Both gene set enrichment (KEGG) terminology and gene ontology (GO) analyses revealed that many of the top gene classes and metabolic pathways are similar to those in longevity interventions reported, suggesting that TCPOBOP may share common molecular pathways regulating longevity with most lifespan extension interventions.

Functional gene set enrichment analysis (GSEA) showed that xenobiotic metabolic process, positive regulation of response to external stimulus, xenobiotic catabolic process, response to xenobiotic stimulus, metabolism of xenobiotics by cytochrome P450, drug metabolism‐cytochrome P450, drug metabolism‐other enzymes were upregulated after TCPOBOP treatment (Figure 8d–j). These data further identify pathways associated with xenobiotic resistance and longevity demonstrate. Specifically, gene expression analysis by RNA‐seq also confirmed that at least 131 of the 1,826 genes up‐regulated by TCPOBOP were targets identified by previous studies as being associated with xenobiotic resistance (Figure 8k). To further identify pathways associated with resistance to xenogeneic stresses and longevity, the expression of differentiate genes was validated by qRT‐PCR and found that TCPOBOP treatment upregulated the genes associated with xenobiotic resistance, including *Cyp1a2*, *Cyp3a11*, *Cyp2c29*, *Cyp2a4*, *Cyp2a5*, *Cyp4b1*, *Cyp2b10*, *Mrp2*, *Mrp3*, *Aldh3a1*, *Aldh2*, *Ces2c*, *Ugt1a1*, and *Gstt1* in the liver of mice (Figure 8j). To further confirm that the expression of xenobiotic resistance genes was increased after TCPOBOP treatment, a western blot was performed to assay the protein levels of Cyp1a1, Cyp3a11, Cyp51a1, and Gsta1. The results showed that the protein levels in the liver were also increased after TCPOBOP treatment (Figure 8m–q). Together, these data suggest that the lifespan‐extending effects of TCPOBOP may be related to xenobiotic resistance regulated by CAR.

### 2.8 TCPOBOP Improves Lifespan and Healthspan in Doxorubicin‐Induced Accelerated Aging

**Figure 9:**
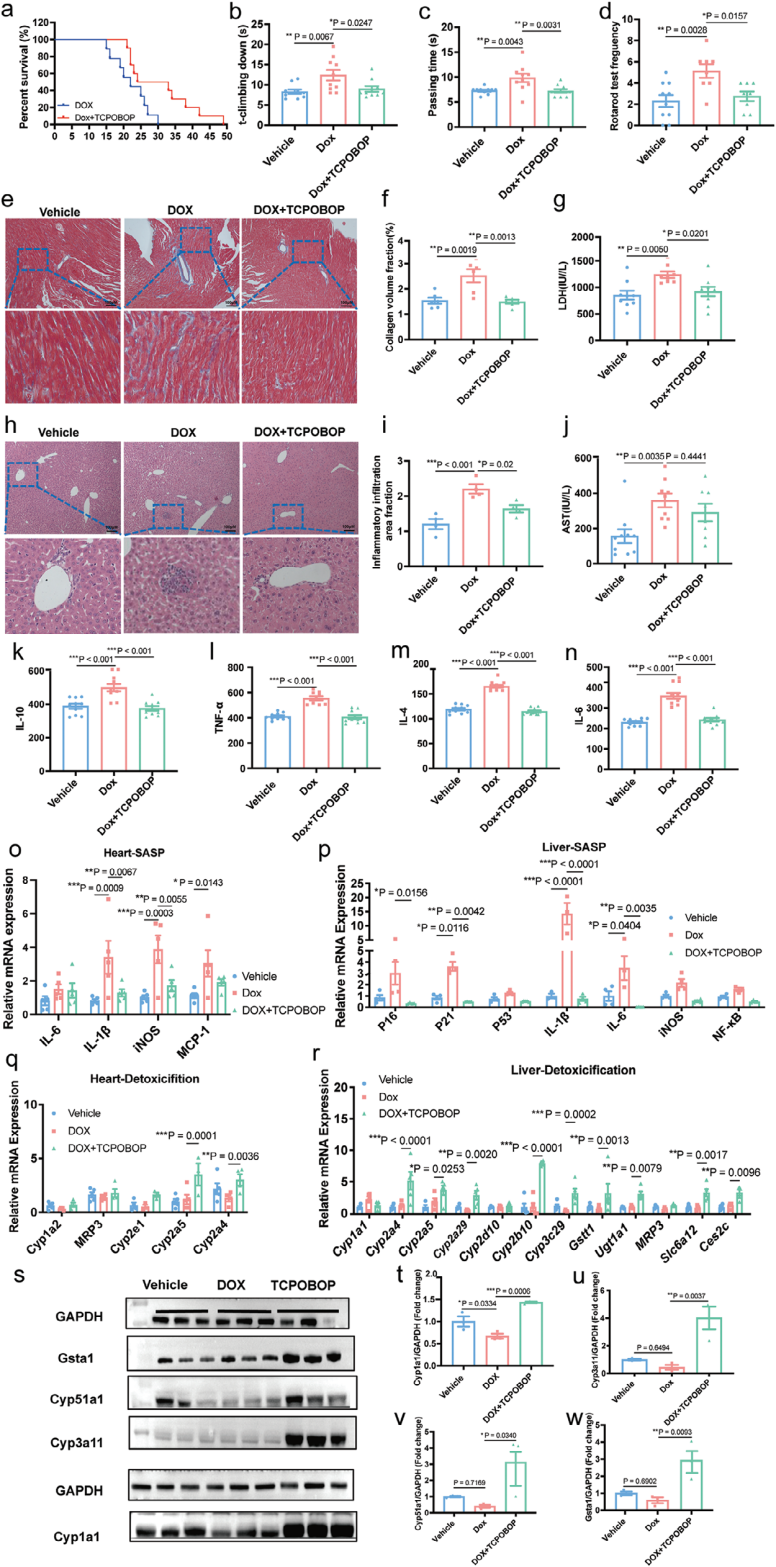
TCPOBOP improves lifespan and healthspan in Doxorubicin‐induced accelerated aging mice. a) The survival curves of doxorubicin‐induced mice. Doxorubicin was administered twice weekly until the last mouse died. The lifespan was analyzed by log‐rank test, and the detailed lifespan analysis was listed in Table S8 (Supporting Information). b) T‐climbing down in the pole test. c) Passing time in the beam balance test. d) Fall of frequencies of the rotarod test (*n* = 8–10 in each group). e) Masson staining of the heart tissue (*n* = 5 in each group). f) The quantification of collagen accumulation in the heart quantified by ImageJ. g) Level of LDH (*n* = 8–10 in each group). h) H&E staining of the liver tissue (*n* = 4 in each group). i) The quantification of inflammatory infiltration in the liver tissue by ImageJ. j) Level of AST (*n* = 8–10 in each group). k–n) SASP inflammatory factor levels in the serum (*n* = 8–10 in each group). k) Level of IL‐10. l) Level of TNF‐α. m) Level of IL‐4. n) Level of IL‐6. o,p) The genes expression related with pro‐aging and SASP in the heart and the liver, *n* ≥ 3 in each group. q,r) The expression of detoxification genes by qRT‐PCR. s) Representative images of Cyp1a1, Cyp3a4, Cyp51a1, and Gsta1 protein, *n* = 3 in each group. t–w) Protein expression quantification of Cyp1a1, Cyp3a4, Cyp51a1, and Gsata1. Data were expressed as mean ± S.E.M. and analyzed by one‐way ANOVA (b–d, f–g, i–n, t–w). Significance was analyzed by Two‐tailed unpaired Student's *t*‐test (o–r). Compared with Dox group, *p < 0.05, **p < 0.01, ***p < 0.001. SASP, Senescence Associated Secretory Phenotype. Dox, doxorubicin.

Chemotherapy drug doxorubicin can lead to accelerated aging and other long‐term health problems in cancer survivors,^[^ ^83^ ^]^ as well as induce cellular and organ senescence in animal models.^[^ ^84^ ^]^ So, this drug has been widely used to induce early aging models. Therefore, we analyzed whether mCAR agonist TCPOBOP can prolong the lifespan and healthy time of doxorubicin‐treated mice. In the lifespan determination, mice were treated with doxorubicin plus TCPOBOP until the last mouse died. Remarkably, the average lifespan of mice treated with TCPOBOP was extended by 31.33% (**Figure** **9**a, Table S8, Supporting Information). In the healthy lifespan experiment, doxorubicin increased the climbing time of the pole and the balance beam (Figure 9b,c), and the number of drops from the rotating bar (Figure 9d), indicating that TCPOBOP can improve the motor ability of premature mice induced by doxorubicin.

Previous studies have shown that doxorubicin has obvious toxic effects on the heart tissues. Prolonged use of doxorubicin can cause severe damage to myocardial cells and induce cardiac hypertrophy and fibrosis, eventually leading to accelerated aging.^[^ ^85^, ^86^, ^87^, ^88^ ^]^ Therefore, we performed Masson staining on mouse heart tissue sections and detected the level of LDH in mouse blood. The results showed that doxorubicin induced myocardial atrophy and collagen deposition in the heart of mice (Figure 9c,d), which is a marker of fibrosis. In addition, compared with the control group, the doxorubicin treatment led to an increase in the LDH level (Figure 9g). After TCPOBOP treatment, the collagen deposition was reduced and the LDH level was decreased (Figure 9e–g), suggesting that TCPOBOP has the protective effect on heart damage. Next, we tested whether TCPOBOP can also improve liver function. Compared with the control group, doxorubicin increased the infiltration of inflammatory cells in the liver and the levels of serum aspartate aminotransferase (AST) and alanine aminotransferase (ALT), which are indicators of liver injury (Figure 9h–j, Figure S3, Supporting Information). Interestingly, the level of ALT was increased after the administration of TCPOBOP (Figure S3, Supporting Information), which might be related to the function of TCPOBOP in inducing hepatic hypertrophy.^[^ ^89^, ^90^ ^]^ However, TCPOBOP counteracted the infiltration of inflammatory cells in the liver and an increase in AST levels (Figure 9e–g). This may be due to the fact that TCPOBOP may cause a transient increase in ALT, but it has not yet developed to an obvious inflammation stage, which is consistent with the literature reports.^[^ ^91^ ^]^

Cellular senescence is the basis of organism aging, and often accompanied by the generation of a senescence‐associated secretory phenotype (SASP). Compared with those in the vehicle group, the serum concentrations of interleukin 10 (IL‐10), tumor necrosis factor‐α (TNF‐α), interleukin 4 (IL‐4), and interleukin 4 (IL‐6) were increased after doxorubicin treatment (Figure 9k–n), while the contents of these cytokines in mice treated with TCPOBOP were decreased (Figure 9k–n), suggesting that TCPOBOP can reduce the level of SASP‐related inflammatory factors. In addition, after doxorubicin induction, the expression of SASP‐related factors such as *MCP‐1*, *iNOS*, and *IL‐1β* were significantly expressed in the mouse heart, while after treatment with TCPOBOP, the expression of *iNOS* and *IL‐1β* decreased significantly (Figure 9o). Meanwhile, in the mouse liver induced by doxorubicin, the mRNA levels of senescence‐related genes *P16*, *P21*, *MCP‐1*, *IL‐6*, and *IL‐1β* were increased significantly. However, after TCPOBOP treatment, the expression levels of the above genes were significantly downregulated (Figure 9p). These results, together with the observed a decrease in the level of SASP‐related inflammatory factors, indicate that TCPOBOP may inhibit SASP during aging process.

To investigate whether TCPOBOP could regulate the xenogeneic gene expression in the liver and the heart of doxorubicin‐treated mice, we carried out qRT‐PCR analysis. The results showed that TCPOBOP upregulated the expression levels of *Cyp2a4* and *Cyp2a5* in the heart (Figure 9q), and *Cyp2a4*, *Cyp2a29*, *Cyp2b10*, *Cyp3c29*, *Gstt1*, *Ugt1a1*, *Slc6a12*, and *CES2C* in the liver (Figure 9r). Meanwhile, Western blot analysis showed that Gsta1, Cyp1a1, Cyp3a4, and Cyp51a1 protein levels were induced by TCPOBOP treatment (Figure 9s–w). Taken together, these data suggest that TCPOBOP may improve the healthspan and extend the lifespan in doxorubicin traeted mice, which may be correlated with the enhancement of xenobiotic resistance.

### 2.9 TCPOBOP Improves Healthspan in SAMP8 Mice

**Figure 10:**
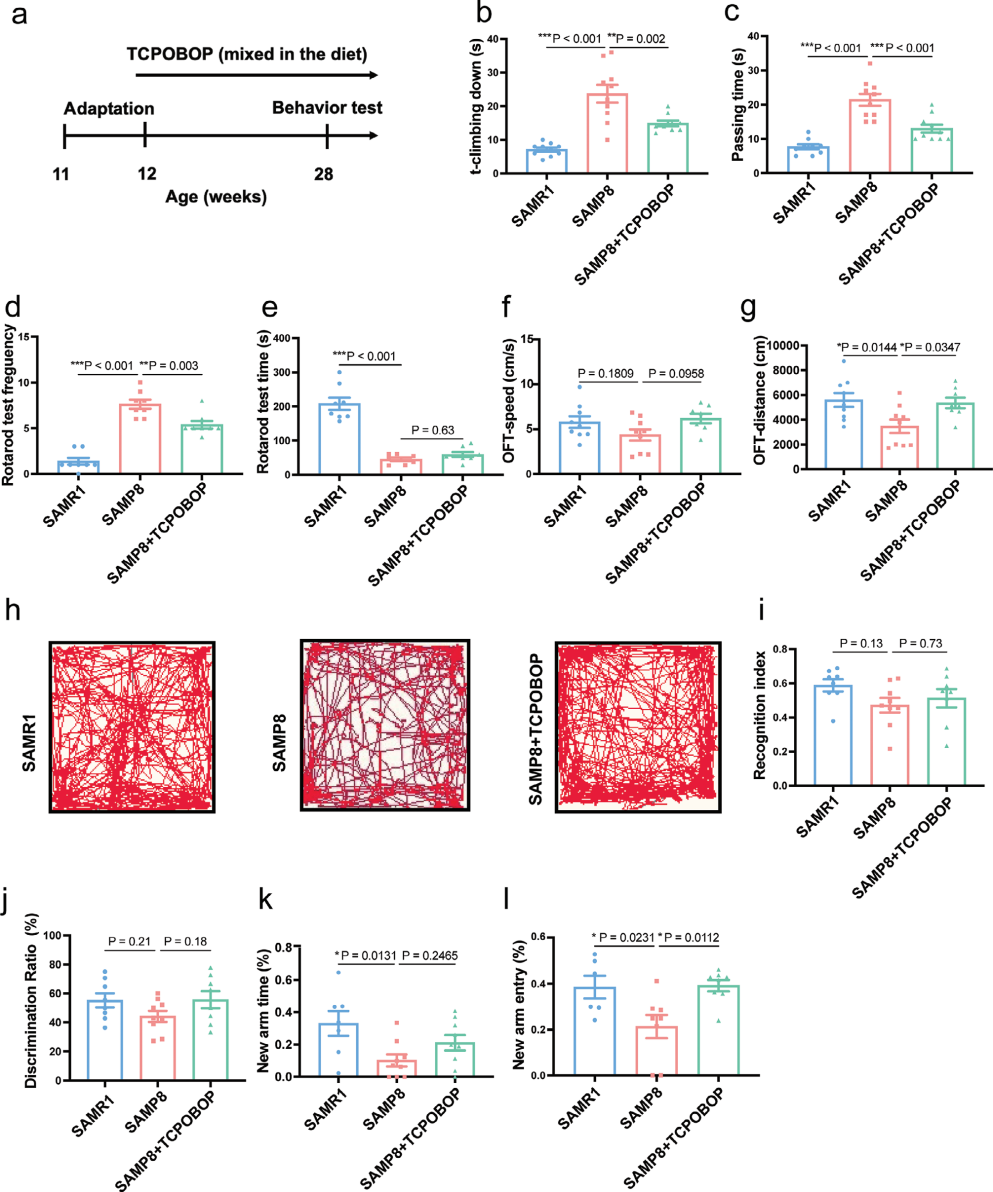
TCPOBOP improves healthspan in SAMP8 mice. a) Timeline for the experimental process. b) T‐climbing down in the pole test. c) Passing time in the beam balance test. d) Fall of frequencies of the rotarod test. e) The time of the first fall of the rotarod test. f) Speed in the center of the open field test. g) Total distance traveled in the open filed test. h) Trajectory in open field. i) Recognition index in novel object recognition test. j) Discrimination ratio in novel object recognition test. k) Time spent in the new arm of the Y maze test. l) Number of entries in the new arm of the Y maze test. The images of mice. Data were expressed as mean ± S.E.M. and analyzed by one‐way ANOVA, *n* = 7–10 in each group. Compared with SAMP8 group, *p < 0.05, **p < 0.01, ***p < 0.001.

SAMP8 (senescence accelerated mice prone 8) has been extensively utilized in senescence studies, mainly attributed to that it shows a deterioration of organs similar to that of older mice after 6 months of age.^[^ ^92^, ^93^ ^]^ To further study the anti‐aging effect of TCPOBOP, we examined the healthspan of SAMP8 mice and used senescence accelerated mouse resistant 1 (SAMR1) mice as the normal control. To further investigate the anti‐aging effects of TCPOBOP, we assayed the healthspan in SAMP8 mice. In contrast to SAMR1 mice, SAMP8 mice displayed an increment in the time expended on T‐climbing and passing the balance beam, an augmentation in the number of falls from the rod, and a decrement in the time spent dwelling on the rod (**Figure** **10**b–e). Additionally, SAMP8 mice tended to downregulate locomotor speed in the center region of the open field (Figure 10f). Nevertheless, these parameters were reversed in SAMP8 mice treated with TCPOBOP (Figure 10b–f). Previous studies have reported mood disorders and memory deficits in SAMP8 mice.^[^ ^94^, ^95^ ^]^ Thus, we employed the open field test to assess anxiety‐like behaviors. Compared to SAMR1 mice, SAMP8 mice showed less frequency of exploring the central region, whereas mice treated with TCPOBOP showed a tendency to an increase in explore the central region (Figure 10g–h). To evaluate the effects of the drugs on the learning and memory capabilities of the mice, we initially performed a novel object recognition experiment. The results revealed that SAMR1 mice displayed a stronger interest in new objects, while SAMP8 mice exhibited a tendency to a decrease in the recognition index. After the TCPOBOP intervention, there was a tendency for the mice to increase their recognition for new objects (Figure 10i–j). Subsequently, we conducted Y‐maze test. The results demonstrated a decreasing in exploration time and the percentage and number of entries into new arms in SAMP8 mice compared with SAMR1 mice, whereas mice treated with TCPOBOP increased the number of entries into new arms (Figure 10k,l). Overall, these findings imply that TCPOBOP may alleviate age‐related disorders such as dyskinesia, anxiety‐like behaviors, and memory deficits in SAMP8 mice.

### 2.10 The Effect of TCPOBOP on Lifespan and Healthspan Depends on CAR in Mice

To further validate the longevity effects of TCPOBOP depend on the activation of CAR, CAR^−/−^ mice were employed to determine the effect of TCPOBOP on the lifespan and healthy lifespan of doxorubicin‐treated mice. Firstly, we conducted lifespan experiments. The results showed that TCPOBOP intervention did not increase the survival rate of CAR^−/−^ mice (Figure S4a and Table S8, Supporting Information). In healthspan analysis, T‐climbing test and balance beam test revealed that TCPOBOP intervention was unable to shorten the time of T‐climbing and passing the balance beam in CAR^−/−^ mice (Figure S4b,c, Supporting Information). These data further suggest that the lifespan‐ and healthspan‐prolonging effects of TCPOBOP occur via CAR signaling in mice.

## 3 Discussion

In the present study, we report that CAR agonists can extend the lifespan and healthspan of *C. elegans* and mice. Given the differences in CAR agonists between humans and mice,^[^ ^96^ ^]^ and our study's dedication to providing clues for the mechanisms of human diseases, the hCAR agonist CITCO was used for the *C. elegans* study, while mCAR agonist TCPOBOP was selected for the mouse study. In this study, CITCO prolonged the average lifespan of *C. elegans* by 12.76% (Figure 1a, Table S1, Supporting Information), while TCPOBOP increased the average lifespan of doxorubicin‐treated mice by 31.33% (Figure 9a, Table S8, Supporting Information). Several lines of evidence support that CAR agonists enhanced healthspan in *C. elegans* and mice. First, CITCO significantly improved the pharyngeal pumping and body bends in nematodes (Figure 2b–e,i). Second, TCPOBOP ameliorated the motor behavior as well as the functions and SASP of the liver and the heart in doxorubicin‐treated mice (Figure 9). Third, TCPOBOP reversed the aging‐related alterations in the skin, liver and central nervous system, and improved the mobility and cognitive dysfunctions of D‐galactose‐treated mice (Figures 6 and 7). Beyond toxicant‐induced aging mouse models, TCPOBOP was capable of enhancing the healthspan of SAMP8 mice, encompassing mobility and cognitive dysfunctions (Figure 10). Most importantly, CITCO was shown to effectively reverse the senescence of HFF‐1 cells induced by D‐galactose and doxorubicin providing compelling evidence of its anti‐aging efficacy (Figure S5, Supporting Information). This finding aligns with observations from *C. elegans* and mouse models, highlighting the conserved and consistent role of CAR across different species. Overall, our study provides the evidence of the role of CAR agonists in extending lifespan and healthspan.

Our results showed that the role of CITCO and TCPOBOP in extending lifespan and healthspan is accomplished through the activation of CAR/NHR signaling in accelerated senescence mice. To begin with, we ascertained that following the administration of CAR agonists, the RNA levels of *nhr‐8/daf‐12* downstream genes, such as *cyp35a2*, *cyp35a5*, *gst‐4*, and *pgps* in nematodes were upregulated. Simultaneously, the RNA levels of CAR target genes, like *Cyp2b10*, *Cyp3a11*, *Cyp2a4*, *Cyp2a5*, Ces2c, *Ugt1a1*, and *Gstt1* in mice were significantly enhanced. Gene expression analysis via transcriptome sequencing (RNA‐seq) also affirmed the activation of multiple downstream target genes of mCAR. Additionally, we observed that after the administration of TCPOBOP, the enrichment of liver differential genes in cellular component, biological process, and molecular function was in congruence with the function of CAR, such as oxidoreductase activity and the response to xenobiotic stimulus in biological processes, and so forth.^[^ ^97^, ^98^ ^]^ Subsequently, we carried out lifespan experiments on *nhr‐8* (*tm1800*) and *daf‐12* (*rh61rh411*) gene mutant nematodes and discovered that CITCO was incapable of extending the lifespan of nematodes. Furthermore, in CAR^−/−^ mice, TCPOBOP failed to increase the lifespan and motor ability following doxorubicin‐treatment, which further supports that the lifespan and healthspan extending effects of CITCO and TCPOBOP may be related to the activation of CAR in mice.

Previous studies have shown that long‐term exposure to environmental stressors may accelerate biological aging.^[^ ^99^ ^]^ The enhanced ability to adapt to stress induced by xenobiotic exposure is a common characteristic of long‐lived *C. elegans*, flies and mice.^[^ ^100^ ^]^ Recently, we reported that targeting PXR‐mediated detoxification might prolong lifespan and healthspan of animals.^[^ ^14^ ^]^ The genes encoded by CAR and PXR regulate xenobiotic metabolizing enzymes with significant overlap.^[^ ^30^ ^]^ In this study, we demonstrated that CAR agonists might increase the resistance of *C. elegans* and mice to xenobiotics, similar to PXR agonists. When *C. elegans* were exposed to MeHgCl, PQ, CC, and CQ, the survival rates of *C. elegans* were decreased significantly, while CAR agonists counteracted the toxic effects of these xenobiotics. Moreover, CAR agonists prolonged the average lifespan of nematodes under H_2_O_2_ stress and heat stress conditions. Interestingly, the xenobiotic resistance effect of CITCO vanished in *nhr‐8* and *daf‐12* mutant *C. elegans*. Simultaneously, CAR agonists reduced the levels of oxidative products ROS and MDA in nematodes, increased the levels of SOD and CAT, and upregulated the expression of xenobiotic stress resistance‐related genes such as anti‐stress and detoxification genes in nematodes and mice. RNA sequencing analysis of mouse livers supported these results, which also indicated that CAR agonists regulated multiple signaling pathways related to xenobiotic resistance. Multiple aging‐related signals may be involved in the lifespan extension and xenobiotic resistance effects of CITCO, such as the insulin/insulin‐like growth factor 1 signaling pathway, AMPK, mTOR, and lipid metabolism, which is agree with the previous findings that CAR may interact with longevity signaling.^[^ ^33^ ^]^ This may be related to the role of CAR in coordinating energy metabolism and immune responses to adverse environmental factors.^[^ ^31^ ^]^ Taken together, the data support that xenobiotic resistance may be a mechanism for promoting longevity of CAR agonists.

The roles of CAR in neurotoxicity induced by xenobiotics (including chemotherapeutic drugs, pesticides and environmental pollutants) have received increasing attention.^[^ ^101^ ^]^ Studies have shown that the regulation of CAR directly enhances the expression of biotransformation transporters and enzymes, potentially providing neuroprotection.^[^ ^31^ ^]^ However, there is no direct evidence to prove that CAR is involved in neuroprotection. Our research demonstrated that CITCO prevented the damage of neurotoxic substances 6‐OHDA and MPP^+^ to the dopaminergic neurons of UA57 nematode (Figure 5g–j), and improved the protein homeostasis in the NL5901 model by reducing the accumulation of α‐synuclein (Figure 5o,p). Besides, CITCO delayed the paralysis rate of the AD model CL4176 and CL2120 strains (Figure 5b,c, Table S6, Supporting Information), and prolonged the lifespan of CL4176. Our study supports the neuroprotective effect of CAR. Interestingly, the improvement effect of CITCO on the paralysis rate of CL4176 is dependent on nhr‐8/daf‐12 signaling. This finding demonstrated the direct involvement of NR in neuroprotection.

The activation of PXR can extend lifespan and healthspan in multiple animal models.^[^ ^14^ ^]^ It is notable that some CAR agonist may also activate PXR. However, it can be excluded that the lifespan‐extending effect of CAR agonists depend on PXR. First, TCPOBOP is a specific mCAR agonist. Second, CITCO displayed >100‐fold selectivity for hCAR over hPXR,^[^ ^102^ ^]^ and CAR silencing can block the anti‐inflammatory and cholesterol metabolism‐regulating effects of CITCO,^[^ ^103^, ^104^ ^]^ implying that CITCO does not activate PXR.

Beyond its established role in enhancing xenobiotic resistance, CAR activation has been identified as a critical regulator of metabolic homeostasis, influencing processes such as lipid, glucose, bile acid metabolism, cell proliferation, tissue regeneration, repair, and cancer development, which are important for maintaining systemic balance during aging.^[^ ^105^ ^]^ In the D‐galactose‐induced aging model, CAR activation was associated with significant transcriptional changes in genes involved in fatty acid and steroid metabolism, contributing to metabolic stability and enhancing the body's resistance to xenobiotics. This could potentially mitigate age‐related decline. Interestingly, in *C. elegans*, the deletion of *fat‐2* and *fat‐3* genes, which encode enzymes essential for the synthesis of unsaturated fatty acids, abolished the lifespan extension induced by CAR activation, highlighting the complex interplay between lipid metabolism and xenobiotic resistance. Furthermore, in the doxorubicin‐induced accelerated aging mouse model, CAR activation alleviated systemic inflammation by reducing pro‐inflammatory cytokines such as IL‐6 and TNF‐α. This reduction in chronic low‐grade inflammation, commonly referred to as “inflammaging,” may help protect against cellular damage associated with aging. Together, these findings underscore CAR's multifaceted role in aging, extending its impact beyond detoxification to include the regulation of metabolic pathways and immune responses. These insights provide valuable perspectives on CAR as a potential modulator of aging and age‐related diseases.

It is important to recognize the limitations of this study, particularly regarding the side effects associated with CAR activation. For instance, it has been linked to hepatomegaly and hepatocyte proliferation, which increases liver stress.^[^ ^106^ ^]^ Another concern is that CAR activation may disrupt the balance of sex hormones and thyroid hormones, leading to endocrine issues.^[^ ^107^ ^]^ Prolonged activation has also been associated with an increased risk of hepatocellular carcinoma.^[^ ^108^, ^109^ ^]^ Notably, TCPOBOP has shown hepato‐protective effects, such as reducing liver injury.^[^ ^110^, ^111^ ^]^ In our study, TCPOBOP reduced liver inflammation, suggesting it might affect an adaptive response rather than toxicity. Emerging evidence from human studies supports CAR complex and context‐dependent role, highlighting its potential hepato‐protective effects under certain conditions.^[^ ^111^ ^]^ These findings underscore the importance of further research to balance CAR therapeutic potential with its risks, paving the way for safe and effective clinical applications.

Of notice, the species‐specific nature of CAR activation may affect the translation of the results. The mouse CAR agonist TCPOBOP and the human CAR agonist CITCO exhibit some difference in their activation properties, which may lead to divergent downstream effects.^[^ ^112^, ^113^ ^]^ Additionally, mice tend to have a stronger induction of detoxification enzymes mediated by CAR than humans, which could limit the clinical efficacy of CAR agonists in human.^[^ ^114^ ^]^ While the accelerated aging models used in this study, such as SAMP8 mice, offer valuable insights into aging mechanisms, they cannot fully replicate the complexity of human aging.^[^ ^115^ ^]^ Similarly, *C. elegans* has distinct physiological and anatomical differences from mammals. Although the molecular mechanisms, such as xenobiotic resistance and key metabolic genes, have homologs in humans, the systemic complexity and tissue‐specific interactions in humans may not be fully captured in these models.^[^ ^14^, ^116^ ^]^ Thus, more study is needed for the translation to clinical application.

To establish a stronger connection between CAR activation and human health, future research should prioritize several key areas. First, larger cohort studies are needed to comprehensively analyze the long‐term effects and safety of CAR activation in humans. Second, a more focused investigation into age‐related diseases such as neurodegenerative disorders and cardiovascular diseases is crucial, as CAR activation may influence the progression of these conditions. Third, there is a pressing need to develop more targeted CAR agonists to enhance efficacy while minimizing potential side effects. These efforts will address critical gaps in our understanding, strengthen the theoretical foundation of CAR‐targeted therapies, and unlock the clinical potential of CAR modulation in aging and related diseases. Ultimately, such research could contribute significantly to improving human health and well‐being.

In conclusion, we found that CAR agonists extended the lifespan and healthspan of *C. elegans* and mice and regulated the gene expression of xenobiotic resistance through the activation of nuclear hormone receptors. The function of CAR in regulating xenobiotic resistance may be related to multiple longevity signaling pathways such as IIS, AMPK, mTOR, and lipid metabolism. Our data suggest that targeting CAR may be a feasible strategy for promoting longevity and health.

## 4 Experimental Section

### Reagents

CITCO (CAS No: 338404‐52‐7) and TCPOBOP (CAS No: 76150‐91‐9) were purchased from Yuanye Biotech (Shanghai, China) with purity >98%. Doxorubicin hydrochloride (Yuanye, Shanghai, China), D‐galactose (Sigma, USA), chloroquine (Yuanye, Shanghai, China), paraquat (Thermo Fisher Scientific, Waltham, USA), colchicine (Yuanye, Shanghai, China), and methylmercury chloride (MeHgCl, Dr. Ehrenstorfer GmbH, Augsburg, Germany) were commercially obtained.

CITCO stock solution was prepared with dimethyl sulfoxide (DMSO) to 50 mm. The working solutions were diluted in *Escherichia coli* OP50 (short as OP50) at final concentrations of 2.5, 5, 10, 20, and 50 µm.

### Strains and Maintenance Conditions

All *C. elegans* were cultured on solid nematode growth medium (NGM) agar plates at 20 °C, which was pre‐seeded with standard food resource of OP50.^[^ ^117^ ^]^ The following strains of *C. elegans* strains were used in this study: N2 Bristol strain (wild type), *daf‐2* (*e1370*), *daf‐16* (*mu86*), DAF‐16::GFP (*muIs109*), *sir‐2.1* (*ok434*), *skn‐1* (*tm4241*), *hsf‐1* (*ps3651*), *aak‐2* (*ok524*), *let‐363* (*ok3018*), *eat‐2* (*ad1116*), *raga‐1* (*ok386*), *fat‐2* (*wa17*), *fat‐3* (*ok1126*), *myo‐3p::Tomm‐20::mKate2::HA (FoxSi16)*, *daf‐12* (*rh61rh411*), *nhr‐8* (*tm1800*), CL4176 [*smg‐1 (cc546)I; dvls27 X*], *CL2120 dvIs14 [(pCL12) unc‐54::beta 1–42 + (pCL26) mtl‐2::GFP]*, NL5901 *((pkls2386)*, and UA57 *(bals4)*. Synchronized worm population was obtained using sodium hypochlorite as previously reported.^[^ ^118^ ^]^

### Lifespan Assay

The synchronized *C. elegans* were transferred to NGM agar plates and incubated at 20 °C unless otherwise noted. Three days later, L4 stage *C. elegans* (day 0 of the longevity assay) were transferred to NGM plates and fed with *E. coil* OP50 supplemented with 10, 20, 50, and 100 µm CITCO or DMSO (0.1%), respectively. *C. elegans* were transferred to a refreshed plate daily during the reproductive period. Once reproduction ceased, the transfer was adjusted to every two to three days. The number of worm deaths was counted when they failed to respond to repeated prodding with picks and showed no signs of pharyngeal pumping. Six plates of 35 worms per plate were tested per assay, and all experiments were conducted independently three times.

For RNA interference (RNAi) lifespan experiments, synchronized L1 stage *C. elegans* were initially fed *E. coli* (HT115) harboring an empty control vector (L4440) until they reached L4 stage. Subsequently, the *C. elegans* were transferred to plates where they were fed with *daf‐12* and *nhr‐8* RNAi constructs, administered with or without CITCO (50 µm). L4440 and DMSO (0.1%) served as controls, with all experiments conducted on NGM plates supplemented with isopropyl‐β‐D‐thiogalactopyranoside (IPTG, 1 mg mL^−1^) and ampicillin (50 µg mL^−1^). All RNAi constructs were obtained from Ahringer RNAi library and were cultured overnight at 37 °C in LB medium containing ampicillin (50 µg mL^−1^) after sequence verification.

### Bacterial Growth Assay

Bacterial growth assays were conducted as previously described.^[^ ^119^, ^120^ ^]^ OP50 was dabbed onto solid LB medium and incubated inverted at 37 °C for 12 h. Immediately thereafter, a clone was picked using a sterile gun tip and transferred to LB medium, where it was incubated at 37 °C for an additional 10 h. CITCO was supplemented in OP50 at the concentrations of 5, 10, 25, 50, and 100 µm, and the OD595 values of OP50 were measured after 0, 2, 4, 6, 8, 10, and 12 h, respectively.

And then the culture supplemented with CITCO for 12 h was serially diluted 250‐fold using LB medium. The diluted samples were then plated onto drug‐containing plates. The inoculated plates were incubated at 37 °C for 16 h. After incubation, visible colonies were observed and counted. The colony‐forming units (CFU) per microliter were calculated using the formula: CFU µL^−1^ = (number of colonies × dilution factor)/inoculation volume.

### Chemotaxis Assay

Initially, 60 mm NGM plates were prepared and inoculated with 20 µL of OP50 supplemented with different concentrations of CITCO (0, 2.5, 5, 10, 25, and 50 µm) at a distance of 0.5 cm from the edge of the plate within the medium sextant. For each concentration, three parallel samples were established. A drop of 10 µL of M9 buffer was placed at the center of the plate, into which 50 early‐stage L4 *C. elegans* were introduced. After M9 buffer was evaporated, the plates were incubated for 4 h at 20 °C. Count the number of *C. elegans* for each concentration hourly (Chemotaxis = *N*_in_/*N*_total_, *N*_in_ = The number of nematodes adhered to the bacterial biofilm in the DMSO or each drug treatment group; *N*_total_ = The total number of nematodes inoculated into the plate). Carry out at least 3 independent experiments for each trial.

### Progeny Production Assay

Ten synchronized wild‐type L4 stage *C. elegans* were transferred to NMG plates with or without CITCO (one nematode per plate) to assess the number of progeny that hatched. The nematodes were transferred to a fresh dish every 24 h until spawning ceased. All plates continued to be incubated in a 20 °C incubator to record the number of eggs hatched.

### Locomotion and Pumping Rate Assays

Synchronized *C. elegans* was cultured as lifespan analysis, and locomotion and pumping rate were recorded as previous methods.^[^ ^121^ ^]^ L4 stage *C. elegans* were treated with DMSO and CITCO (50 µm), respectively. The number of pharyngeal pumps and sinusoids formed in 30 s were observed and counted under a light microscope on days 3, 5, 7, 9, 11, 13, and 15 of adulthood. In addition, the motility of *C. elegans* was assessed and the locomotor behavior of each group was recorded on days 9, 11, 13, and 15 of adulthood. Based on the locomotor status of the nematodes, three grades were classified: A (energetic, symmetrical, and spontaneous locomotion), B (rigid, uncoordinated locomotion, usually requiring stimulation), and C (movement restricted to head or tail in response to a stimulus).

### Mitochondrial Integrity Analysis

The *myo‐3p::Tomm‐20::mKate2::HA* (FoxSi16) nematodes were synchronized and treated with CITCO. On days 7 and 14, they were placed on 2% agarose pads. After these manipulations, the integrity of mitochondria was observed under a German Leica laser scanning confocal microscope. For each group, at least ten nematodes were observed. The fluorescence intensity of somatic mitochondria was quantified using ImageJ software.

### Aging‐Related Neurodegenerative Disease Assays

The UA57 strain and the wild‐type N2 strain were grown synchronously at 20 °C to the L1 stage. ≈400 nematodes were then transferred to a new 1.5 mL EP tube and centrifuged at 3500 rpm for 3 min. The supernatant was discarded, and the nematodes were washed three times with M9 buffer and resuspended. The drug was added, and the nematodes were incubated for 30 min, followed by a 1‐h incubation with 6‐OHDA (10 mm) in the dark. Washed three times again and transferred to new NGM plates containing the drug. The blank and positive control groups were treated with DMSO and cabergoline (Caber), respectively. After 9 days of drug treatment, the nematodes were transferred to NGM plates containing OP50 and after 10 s of acclimatization, their locomotion was observed. Subsequently, the nematodes were placed on 2% agarose pads and examined using a German Leica laser scanning confocal microscope. The fluorescence intensity of the nematode head was quantified using ImageJ. Similarly, strain NL5901 was grown synchronously at 20 °C to the L1 stage. The nematodes were then transferred to a new NGM containing the drug. After seven days of drug administration, the nematodes were transferred to a 2% agarose gel pad with 2% agarose and observed under a German Leica laser scanning confocal microscope. The expression of α‐synuclein in the muscle tissue of the body wall of NL5901 nematodes was quantified using ImageJ. The blank and positive control groups were treated identically with DMSO and cabergoline, respectively.

Strain *CL4176* was synchronized and treated with CITCO from the L1 to L3 at 16 °C, and then the *C. elegans* were placed at 25 °C. The state of the *C. elegans* was observed and recorded daily using a stereomicroscope. *C. elegans* were classified as exhibiting pathological paralysis if they were unable to crawl forward or backward when lightly touched with a platinum wire. The number of paralyzed *C. elegans* was determined accordingly, and the paralysis curve was plotted using GraphPad Prism 10.

### Stress Resistance Assays

In the stress resistance analysis, ≈50 N2 *C. elegans* were monitored following exposure to the indicated stressors. Before exposure to the stressors, L4 stage N2 *C. elegans* were treated with CITCO for 5 days. During the heat shock assay, the *C. elegans* were incubated at 35 °C and observed every 2 h until all *C. elegans* died. For the oxidative stress assay, the *C. elegans* were transferred to new NGM plates mixed with 8 mm H_2_O_2_ at 20 °C and monitored hourly until all *C. elegans* died.

### Detoxification Assay

Paraquat (PQ, 200 µm), methylmercury chloride (MeHgCl, 2 µm), colchicine (CC, 4 mm) and chloroquine (CQ, 4 mm) solutions were prepared with M9 buffer or S buffer, filtered through 0.22 µm nitrocellulose filters, and mixed with varying concentrations of CITCO to achieve final concentrations of 0, 10, 25, 50, and 100 µm. The solutions were then dispensed into 96‐well plates at 100 µL per well. Synchronized L4 stage *C. elegans* were treated with different concentrations of CITCO for 6 days. They were then transferred to a poison mixture containing the same concentration of CITCO, with 5 *C. elegans* per well in a 96‐well plate. The *C. elegans* were incubated for the specified time points and monitored under a stereomicroscope.

### Antioxidant Capacity Assay

The total antioxidant capacity of CITCO was detected using a Total Antioxidant Capacity Assay Kit with ABTS method (Biyun Tian, China). The total antioxidant capacity of CITCO was calculated by detecting the absorbance at 414 nm using 3‐ethylbenzthiazoline‐6‐sulfonic acid (ABTS) as the chromogenic agent. Glutathione was used as positive control.

The assay of antioxidant capacity of *C. elegans* was established according to the described protocol.^[^ ^122^ ^]^ The reactive oxygen species (ROS) level in *C. elegans* was measured with 2,7‐dichlorodiacetate fluorescein (DCFH‐DA) to determine the antioxidant capacity. After L4 stage, N2 *C. elegans* were treated with CITCO (50 µm) for 5 d. About 2000 *C. elegans* were collected and washed completely with M9. Subsequently, the *C. elegans* were sonicated for 30 s and centrifuged at 4 °C for 5 min. Next. 50 µL of the supernatant was mixed with 50 µL of 100 µm H2DCF‐DA solution. Finally, the fluorescence intensity was measured at 530 and 485 nm emission/excitation wavelengths using a microplate reader (BioTek Instruments).

For intracellular malondialdehyde (MDA) content, as well as superoxide dismutase (SOD) and catalase (CAT) activities, L1 stage N2 *C. elegans* were treated with CITCO until the *C. elegans* had grown to L4 stage, and the *C. elegans* were washed completely with M9 buffer and ground for 120 s with a High‐Throughput Tissue Grinder (SCIENTZ, China). The suspensions were centrifuged according to the instructions of the ELISA kit (Biyuntian, China) and the supernatants were extracted for the determination of SOD, CAT, and MDA. Protein concentration was determined using a BCA kit (Biyuntian, China) to standardize the enzyme activities.

### DAF‐16::GFP Translocation Experiments

GFP‐labeled reporter strains of *daf‐16* were treated with CITCO (50 µm) from L1 to L4 in *C. elegans*, anesthetized with 100 mm NaN3 and transferred to slides coated with 2% agarose pads. Ten animals were randomly selected to observe GFP fluorescence signals indicative of DAF‐16 localization under a German Leica laser scanning confocal microscope, and the number of GFP‐positive nuclei was counted for each worm.

### Quantitative Real‐Time Polymerase Chain Reaction (RT‐PCR) Assay

To conduct gene expression analysis, L4 stages treated with 50 µm CITCO were washed with M9 buffer, and then total RNA of *C. elegans* was extracted by Trizol reagent (Vazyme, Nanjing, China) according to the manufacturer's instructions. The extracted total RNA served as a template for reverse transcription into complementary DNA (cDNA) using a cDNA synthesis kit (Vazyme, Nanjing, China) in conjunction with an ABI StepOnePlus Real‐Time Polymerase Chain Reaction System (Applied Biosystems, Foster City, CA, USA). Quantitative real‐time PCR was performed using a SYBR Green PCR Master Mix (Shanghai Sangong Biotechnology Co., Ltd.). The mRNA levels of the genes were measured with *β‐actin* as an internal reference. The sequences of all primers are listed in Table S9 (Supporting Information).

In mouse experiments, total RNA was extracted from 10 mg of tissue and quantitative PCR was performed as described above. The primer sequences are provided in Table S10 (Supporting Information).

### Doxorubicin Induced Accelerated Aging Mice

All the procedures involving animals in this study were approved by Shanghai University of Traditional Chinese Medicine (Approval Number: PZSHUTCM2303130010). All mice were housed under a constant temperature ranging from 22 to 23 °C, and in a 12‐h light and 12‐h dark cycle, with unrestricted access to food and water. The animals acclimated to their new environment for one week prior to the commencement of the experiment. Doxorubicin was utilized to induce senescence in mice. Male C57BL/6J mice (8 weeks) received intraperitoneal injections of doxorubicin at a dose of 5 mg kg^−1^ body weight. The control group mice were injected with an equivalent volume of saline. For lifespan, the mice were continued receiving doxorubicin (5 mg kg^−1^) twice weekly and TCPOBOP (1 mg kg^−1^ day^−1^) until all mice died. For healthspan experiments, the mice were received doxorubicin (5 mg kg^−1^ two times per week) intraperitoneally for 2 weeks and TCPOBOP (1 mg kg^−1^ day^−1^) through gavage for 4 weeks. At the end of healthspan experiments, the mice were performed the pole test and balance beam. Then the mice were euthanized, and the blood and tissues were collected for further analysis.

CAR null knockout mice (C57 BL/6JGpt‐*Nr1i3^em28Cd6919^* /Gpt) were obtained from GemPharmatech Co., Ltd. (Jiangsu Province, China). For more information about this mouse, please visit *https://cn.gempharmatech.com/shop/productDetails/62978*. Twelve mice, consisting of both male and female individuals, were categorized into two groups based on age: the doxorubicin group and the TCPOBOP group, with six mice in each group. Senescence induction using doxorubicin and TCPOBOP treatment were carried out as previously described. Concurrently, wild‐type mice were utilized as the control group. Two weeks after the administration of doxorubicin, the pole test and balance beam were examined as mentioned earlier.

### D‐Galactose‐Induced Pre‐Aging Mice

Thirty male C57BL/6 mice aged 8 weeks were obtained from Beijing Vital River Laboratory Animal Technology Co., Ltd. The animals were categorized into 3 groups: the control group, the D‐galactose (300 mg kg^−1^) group, and the group of D‐galactose‐induced mice treated with TCPOBOP (6.67 mg per 1 kg of food), each consisting of 10 mice. Following eight weeks of continuous treatment with D‐galactose and TCPOBOP, behavioral tests were carried out. At the termination of the animal experiment, the mice were fasted throughout the night, narcotized with 20% urethane, and cardiac blood and tissues were gathered for further analysis.

### SAMP8 Mice

Nineteen male SAMP8 mice and nine male SAMR1 mice were procured from Beijing HFK Bioscience, China. The SAMP8 mice were randomly categorized into two groups based on their body weight. TCPOBOP was included in the diet at a concentration of 6.67 mg per 1 kg of food, commencing at 12 weeks of age. Subsequently, when the mice reached 28 weeks of age, they were selected to have their behaviors tested. Body weight and food intake were registered every two days.

### Histological Analysis

On the final day of behavioral testing, mice were deprived of food overnight and anesthetized by means of 20% urethane, and then the cardiac blood, liver, heart and brain tissues were gathered. For histological examination, the liver, heart and brain tissues were fixed in 4% formalin solution, embedded in paraffin, and sliced at 5 µm. These sections were stained with hematoxylin‐eosin (HE), Masson and TUNEL staining in line with standard protocols. Moreover, fresh liver tissues were prepared for frozen sections and stained with SA‐β‐gal as per established procedures. The morphology was observed using a microscope (Zeiss, Germany). ImageJ was utilized for quantifying the images.

### Behavioral Assessment

The pole test was conducted to assess motor function. In brief, each mouse was positioned on the small ball (with a diameter of 2.5 cm), with its head facing forward. The time it took for the mouse to turn on the ball and descend from a pole (with a diameter of 1 cm and a height of 52 cm) was recorded. Before the test, the mice underwent three training sessions, followed by two testing sessions, and the data from the two tests were averaged. The balance beam test was carried out to measure coordination and balance. A cylindrical wooden stick (with a diameter of 1 cm and a length of 50 cm) was fixed on the cage using medical tape. The mice were placed at one end of the stick, and the time it took for each mouse to reach the other end was noted. Each mouse received three pre‐training sessions and then was tested twice. If a mouse took more than 30 s, the maximum passing time was recorded as 30 s. The rotarod test was measured to evaluate motor coordination and balance.^[^ ^123^ ^]^ Briefly, the mouse was trained, and the speed (rpm) was slowly increased from 5 to 40 at a uniform rate. The time and frequency of falls from the rotarod rod were documented.

The Open Field test is a useful approach for evaluating locomotor activity and emotional responses.^[^ ^124^ ^]^ Briefly, the mice were placed individually into a black open field tank (50 × 50 × 50 cm), with each mouse gently placed in the center. The behavior was recorded for 15 min. The time spent traveling in the center area of the open field and the total traveling distance were analyzed by a video‐tracking system (Noldus Information Technology, Leesburg, VA).

The elevated plus maze test was used to assess anxiety‐related behavior as described before.^[^ ^125^ ^]^ The maze comprised two open arms (30 × 5 cm), two closed arms (30 × 5 × 15 cm), and a central area (5 × 5 cm), all raised 60 cm above the floor. Each mouse was placed in the center area facing an open arm and permitted to explore freely for 15 min. The time spent and the entries into both the open and closed arms were recorded independently.

The Y‐maze test was utilized for evaluating spatial memory ability.^[^ ^126^ ^]^ It comprises a start arm, a secondary arm, and a novel arm (blocked during the training session and opened during the test). The angle among each arm is 120°. During the training session, the mouse was freely enabled to explore the two arms (start arm and secondary arm) for 10 min. After 2 h, each mouse was placed again in the maze at the start arm and given permission to explore all three arms for an additional 10 min. The time spent and the number of entries into the novel arm were documented.

The novel object recognition task is utilized to evaluate the working memory.^[^ ^127^ ^]^ The test was performed in a white box sized 50 × 50 × 50 cm. During the training stages, two indistinguishable objects marked as “A” were positioned in the chamber, and the mouse was allowed to investigate these objects for 5 min. Exploration was determined when the mouse's head was oriented toward the object with its nose within 2 cm of it. Two hours later, one of the objects “A” was swapped with “B” and the procedure was carried out as stated earlier. After 24 h, the remaining object “A” was replaced with “C.” The time and frequency of exploration for both objects (novel and familiar) were recorded for analysis.

### Senescence β‐Galactosidase Staining

HFF‐1 cells were cultured in DMEM supplemented with 15% FBS and seeded at a density of 1 × 105 cells per well in 6‐well plates. The cells were treated with 50 mg mL^−1^ D‐galactose and 5 µm Doxorubicin for 24 h. Following the treatment, the cells were exposed to increasing concentrations of CITCO (1, 2.5, 5, and 10 µm) for an additional 24 h. β‐Galactosidase staining was performed using the Senescence β‐Galactosidase Staining Kit (Shanghai Biyuntian Biotechnology Co., Ltd.), after which images were captured, and the area of positive staining was quantified.

### Statistical Analysis

All tests were statistically analyzed using GraphPad Prism 10.0 (GraphPad Software Inc., San Diego, CA, USA) and SPSS (version 21.0). Lifespan test results were analyzed using Kaplan–Meier survival analysis, and between‐group comparisons were scored for significance using the log‐rank test.  Data between the two groups were compared using Student's *t*‐test. Values have been expressed as ± SEM. Statistical analysis was performed using SPSS statistical software. *p*‐values less than or equal to 0.05 were considered statistically significant.

## Conflict of Interest

The authors declare no conflict of interest.

## Supporting information



Supporting Information

## Data Availability

All data generated or analyzed during this study are included in this published article.
