# Supplementary material for: Activation of Nuclear Receptor CAR: A Pathway to Delay Aging through Enhanced Capacity for Xenobiotic Resistance
Source: Adv Sci (Weinh). 2025 Jan 31;12(12):2416823. doi: 10.1002/advs.202416823 (PMC11948022; doi:10.1002/advs.202416823)
Supplement: Supplementary file 1 — Supporting Information [file ADVS-12-2416823-s001.pdf]

ADVANCED SCIENCE
Open Access

Supporting Information

for *Adv. Sci.*, DOI 10.1002/advs.202416823

Activation of Nuclear Receptor CAR: A Pathway to Delay Aging through Enhanced Capacity for Xenobiotic Resistance

Jing Yu, Xiaoyan Gao, Hang Shi, Lijun Zhang, Wenlong Nie, Ruochen Zhang, Minglv Fang, Ying Liu, Yingxuan Yan, Bingbing Fan, Chengyuan Wu, Cheng Huang and Shengjie Fan**

Supplementary Information for

Activation of Nuclear Receptor CAR: A Pathway to Delay Aging through Enhanced Capacity for Xenobiotic Resistance

Jing Yu[1, 2], Xiaoyan Gao[1, 2], Hang Shi[1], Lijun Zhang[1], Wenlong Nie[1], Ruochen Zhang[1], Minglv Fang, Ying Liu, Yingxuan Yan[1], Bingbing Fan[1], Chengyuan Wu[1], Cheng Huang[1]*, Shengjie Fan[1]*

Affiliations:

1 School of Pharmacy, Shanghai University of Traditional Chinese Medicine, Shanghai, 201203, China

2 These authors contributed equally: Jing Yu, Xiaoyan Gao

*Correspondence: Cheng Huang: chuang@shutcm.edu.cn; or Shengjie Fan: shengjiefan@shutcm.edu.cn

This PDF file includes:

- Supplementary Fig.S1 to S5
- Supplementary Tables 1 to 9
- Supplementary Results

Supplementary Fig. S1

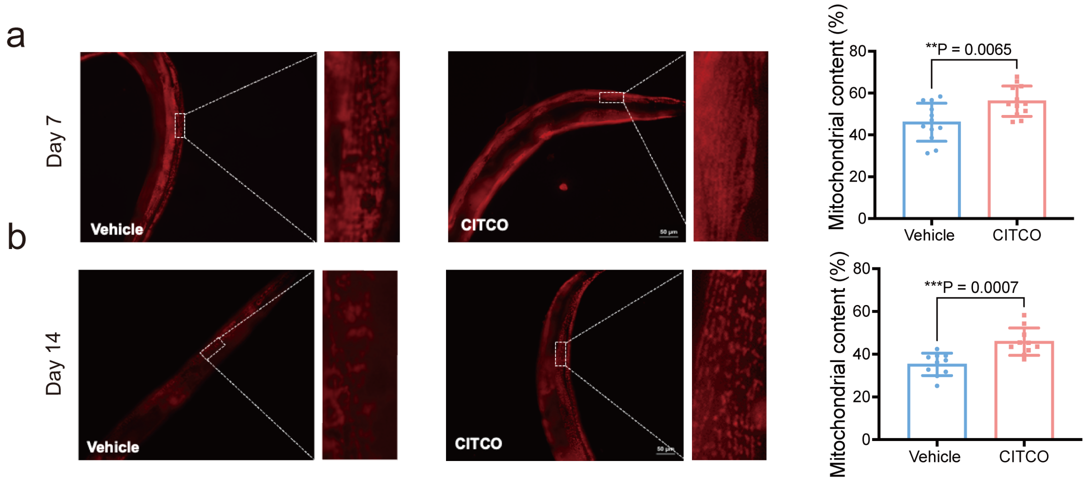

Supplementary Fig. S1. CITCO protects mitochondrial integrity. (a) Representative images and RFP quantification of mitochondrial content at day 7 of adulthood in muscle (pmyo-3::mtRFP) mito::RFP reporter strains (n ≥10). Scale bar, 50 µm. (b) Representative images and RFP quantification of mitochondrial content at day 14 of adulthood in muscle (pmyo-3::mtRFP) mito::RFP reporter strains treated with CITCO or vehicle (n ≥10). Scale bar, 50 µm.

Supplementary Fig. S2

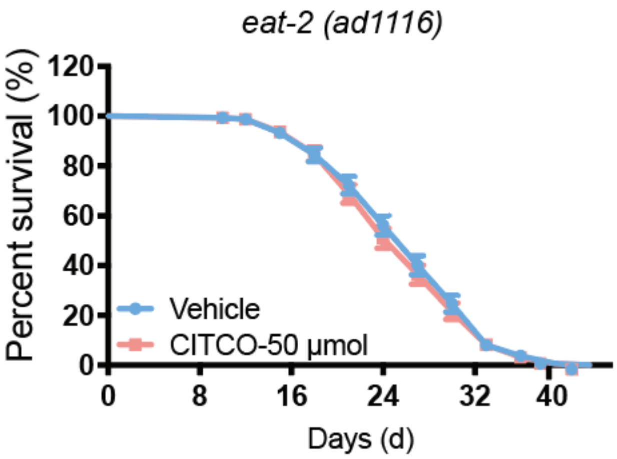

Supplementary Fig. S2. CITCO can not extend lifespan in *eat-2* mutant *C. elegans*. Survival curves of *eat-2 (ad1116)* treated with CITCO (log-rank test, each involving >157 animals).

Supplementary Fig. S3

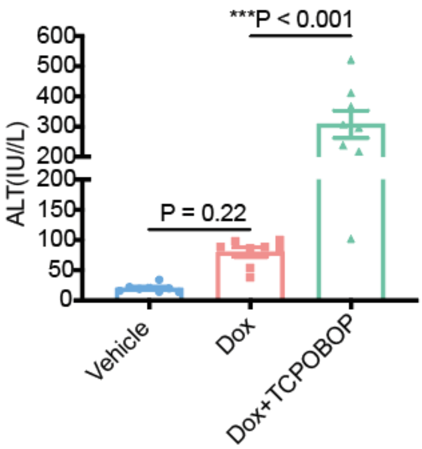

Supplementary Fig. S3. Effects of TCPOBOP on the liver function of mice treated by Dox. Level of ALT in Dox-induced mice (*n*=8).

Supplementary Fig. S4

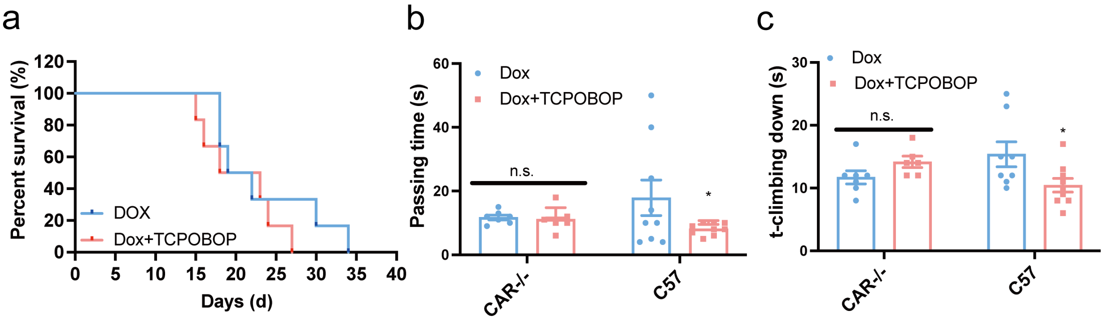

Supplementary Fig. S4. The effect of TCPOBOP on lifespan and healthspan is dependent on CAR. (a) The survival curve of $CAR^{-/-}$ mice. For lifespan assay, the mice were administrated intraperitoneally with doxorubicin at dose of 5 mg/kg body weight twice weekly. The experiment was concluded when the last mouse died. The detailed lifespan analysis by long-rank test was listed in Supplementary Table S8. (b) The passing time on the balance beam in WT mice and $CAR^{-/-}$ mice. (c) The time of climbing down from the pole test in WT mice and $CAR^{-/-}$ mice. Data are expressed as mean ± SEM. Compared with doxorubicin group, * $p < 0.05$; Compared with WT mice group.

Supplementary Fig. S5

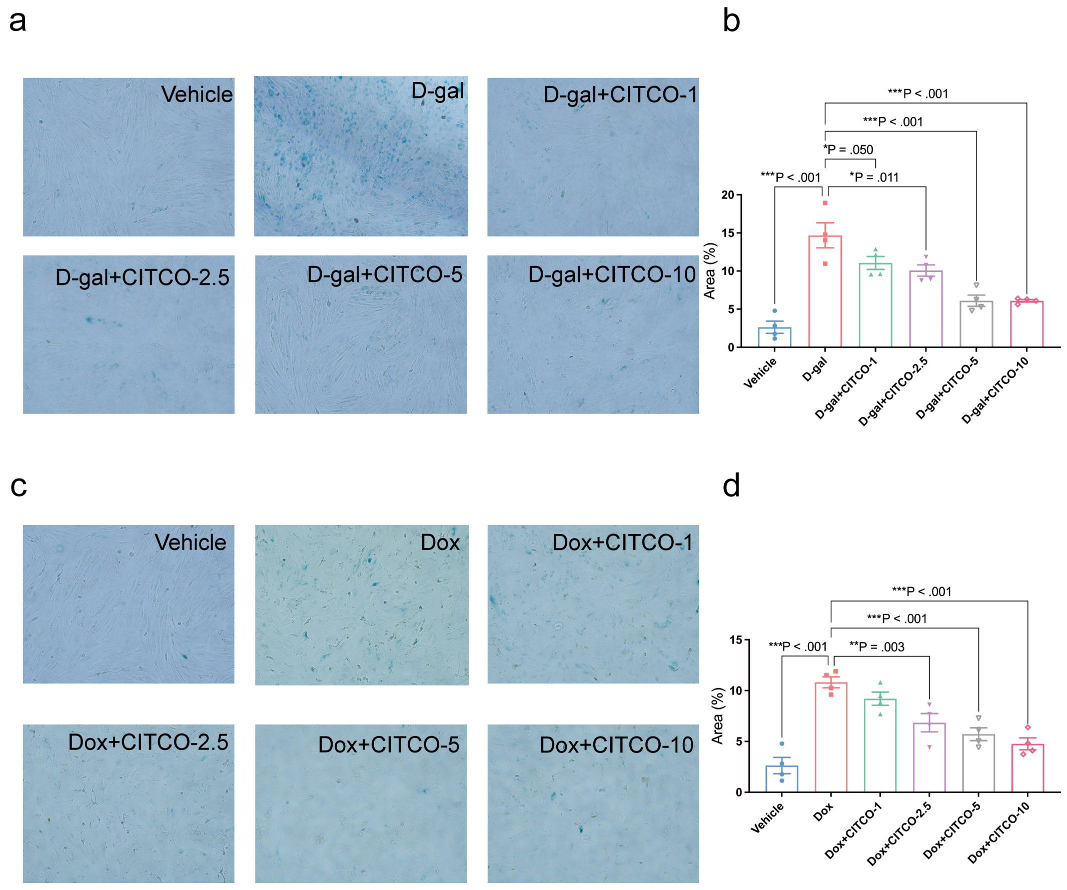

Fig. S5 CITCO can delay the senescence of HFF-1 cells induced by doxorubicin and D-galactose. (a) SA-β-gal staining of human foreskin fibroblasts (HFF-1) after treatment with 50 mg/ml D-gal. (b) Statistics on the positive area of SA-β-gal staining of HFF-1 treated with 50 mg/ml D-gal (c) SA-β-gal staining of human foreskin fibroblasts (HFF-1) after treatment with 5 μmol Doxorubicin. (d) Statistics on the positive area of SA-β-gal staining of HFF-1 treated with 5 μmol Doxorubicin. The concentration unit of CITCO is μmol. Significance was analyzed by two-way ANOVA. All data were presented as mean ± S.E.M. Compared with vehicle group in (a-b), compared with D-gal group in (c) and compared with Doxorubicin group in (d), * p <0.05, ** p < 0.01, *** p < 0.001. D-gal, D-galactose; Dox, doxorubicin.

Supplementary Table S1. Effects of CITCO on the lifespan of N2 *C. elegans*

Group	Number	Mean Survival ± SEM (days)	Median Survival± SEM (days)	Increase (%)	*p* value
Vehicle	138	19.109±0.386	20.00±0.503	/	/
CITCO-10 μM	175	21.274± 0.330	20.00± 0.339	11.32	<0.001***
CITCO-25 μM	128	21.226± 0.400	23.00 ± 0.398	11.29	<0.001***
CITCO-50 μM	146	21.548± 0.376	23.00± 0.286	12.76	<0.001***
CITCO-100 μM	184	21.299± 0.351	20.00 ± 0.326	11.46	<0.001***

Notes: Lifespan experiments were analyzed using Kaplan-Meier survival analysis and compared among groups, scoring for significance using the log-rank test. All data were expressed as mean± SEM. ***$p < 0.001$ *vs.* vehicle.

Supplementary Table S2. Effects of CITCO on the lifespan of N2 *C. elegans* under conditions of 35°C and H_2O_2.

Strain	Type	Group	Number	Mean Survival ± SEM （hours）	Maximum longevity (hours)	Median survival time (hours)	Increase （%）	*p* value
N2	H_2O_2	Vehicle	61	3.98±0.16	7	4	/	/
		CITCO-50 μM	68	4.97±0.17	8	5	24.87	<0.001***
	35℃	Vehicle	69	6.37±0.28	12	8	/	/
		CITCO-50 μM	58	7.91±0.24	14	10	24.18	<0.001***

Notes: Lifespan experiments were analyzed using Kaplan-Meier survival analysis and compared among groups, scoring for significance using the log-rank test. All data were expressed as mean± SEM. ***$p < 0.001$ *vs.* vehicle.

Supplementary Table S3. Effects of CITCO on the lifespan of N2, *nhr-8* and *daf-12* *C. elegans*

Strain	Group	Number	Mean Survival ± SEM (days)	Maximum longevity (days)	Median survival time (days)	Increase (%)	*p* value
N2	Vehicle	162	19.241±0.466	33	19	/	
	CITCO-50 μM	168	21.506±0.442	35	22	11.77	<0.001***
nhr-8	Vehicle	244	18.041±0.289	29	17	/	
	CITCO-50 μM	269	17.959±0.162	29	17	-2.40	n.s.
daf-12	Vehicle	172	18.040±0.313	28	19	/	/
	CITCO-50 μM	212	17.315±0.357	28	16	-4.02	n.s.

Notes: Lifespan experiments were analyzed using Kaplan-Meier survival analysis and compared among groups, scoring for significance using the log-rank test. All data were expressed as mean± SEM. ***$p < 0.001$ *vs.* vehicle.

Supplementary Table S4. Effects of CITCO on the lifespan of *nhr-8* and *daf-12 C. elegans* under conditions of 35°C and H_2O_2.

Strain	Type	Group	Number	Mean Survival ± SEM (hours)	Maximum longevity (hours)	Median survival time (hours)	Increase (%)	*p* value
nhr-8	H_2O_2	Vehicle	64	3.969±0.136	6	4	/	
		CITCO-50 μM	68	3.691±0.119	6	4	-2.40	n.s..
	35℃	Vehicle	66	10.242±0.424	16	10	/	n.s.
		CITCO-50 μM	66	8.636±0.436	16	10	-15.68	0.015*
daf-12	H_2O_2	Vehicle	70	4.500±0.199	6	3	/	/
		CITCO-50 μM	54	3.444±0.103	6	3	-23.47	<0.001***
	35℃	Vehicle	62	9.097±0.337	14	8	/	/
		CITCO-50 μM	65	9.200±0.266	14	10	1.13	n.s.

Notes: Lifespan experiments were analyzed using Kaplan-Meier survival analysis and compared among groups, scoring for significance using the log-rank test. All data were expressed as mean± SEM. *$p < 0.05$, ***$p < 0.001$ vs. vehicle.

Supplementary Table S5. Effects of CITCO on the lifespan of N2, *daf-2*, *age-1*, *daf-16*, *skn-1*, *hsf-1, sir2.1*, *let363, raga-1, aak-2, fat-2, fat-3* and *eat C. elegans*

Strain	Group	Number	Mean Survival ± SEM （hours）	Maximum longevity (days)	Median survival time (days)	Increase （%）	*p* value
N2	Vehicle	130	17.354±0.468	28	17	/	/
	CITCO-50 μM	129	20.085±0.436	31	19	15.74	<0,001***
daf-2	Vehicle	197	43.569±0.558	59	44	/	/
	CITCO-50 μM	178	41.983±0.608	59	44	-3,64	n.s.
age-1	Vehicle	200	27.770±0.525	43	28	/	/
	CITCO-50 μM	211	26.368±0.499	43	28	-2.11	n.s.
daf-16	Vehicle	226	15.664±0.251	25	14	/	/
	CITCO-50 μM	253	14.779±0.224	25	14	-5.65	n.s.
skn-1	Vehicle	211	17.679±0.301	30	16	/	/
	CITCO-50 μM	215	17.995±0.284	30	19	1.79	n.s.
hsf-1	Vehicle	127	11.648±0.313	21	15	/	/
	CITCO-50 μM	107	12.747±0.129	21	12		n.s.
sir-2.1	Vehicle	186	21.419±0.458	33	21	/	/
	CITCO-50 μM	168	17.952±0.481	30	21	-16.19	<0.001***
let363	Vehicle	130	18.938±0.454	32	19	/	/
	CITCO-50 μM	123	18.577±0.474	29	19	-1.91	n.s.
raga-1	Vehicle	180	24.656±0.464	40	24		/
	CITCO-50 μM	178	22.786±0.408	37	21	-7.58	n.s.
aak-2	Vehicle	149	16.819±0.310	24	17	/	/
	CITCO-50 μM	150	16.719±0.314	24	17	-0.595	n.s.
fat-2	Vehicle	50	19.880±0.651	28	22		
	CITCO-50 μM	85	18.282±0.480	28	19	-8.04	n.s.
fat-3	Vehicle	202	14.196±0.375	26	15	/	
	CITCO-50 μM	158	13.361±0.500	23	13	-5.89	n.s.
eat	Vehicle	162	26.451±0.501	42	27	/	
	CITCO-50 μM	157	26.000±0.500	42	27	-1.71	n.s.

Notes: Lifespan experiments were analysed using Kaplan-Meier survival analysis and compared among groups, scoring for significance using the log-rank test. All data were expressed as mean± SEM. ***$p < 0.001$ *vs* vehicle group.

Supplementary Table S6. Effects of CITCO on paralysis of *CL4176* and *CL2120* induced by Aβ

Strain	Group	Paralysis number	Mean paralysis-free time± SEM (hours)	Maximum paralysis-free time (hours)	Median paralysis-free time (hours)	Increase (%)	*p* value
CL4176	Vehicle	172	40.378±0.345	48	40	/	/
	CITCO-50 μM	117	43.786±0.312	50	43	8.44	< 0.001***
CL2120	Vehicle	119	66.958±3.349	138	72	/	
	CITCO-50 μM	115	92.661±2.885	144	96	38.39	< 0.001***
CL4176 (L4440)	Vehicle	131	59.611±0.417	70	60	/	
	CITCO-50 μM	118	63.051±0.455	73	64	5.77	< 0.001***
CL4176 (*daf-12 RNAi*)	Vehicle	110	61.018±0.434	70	60	/	
	CITCO-50 μM	108	58.917±0.367	70	60	-1.8	< 0.001***
CL4176 (*nhr-8 RNAi*)	Vehicle	110	57.409±0.494	67	57	/	
	CITCO-50 μM	82	56.524±0.498	67	57	-1.54	n.s.

Notes: Survival analysis of paralysis were used Kaplan-Meier survival analysis and compared among groups, scoring for significance using the log-rank test. All data were expressed as mean± SEM. ***$p < 0.001$ vs. control group.

Supplementary Table S7. Effects of CITCO on the lifespan *CL4176 C. elegans*

Strain	Group	Number	Mean Survival ± SEM （days）	Maximum longevity (days)	Median survival time (days)	Increase （%）	*p* value
CL4176	Vehicle	207	5.717±0.083	5.5	11	/	
	CITCO-50 μM	202	6.344±0.102	6	12	10.97	<0.000.

Notes: Lifespan experiments were analyzed using Kaplan-Meier survival analysis and compared among groups, scoring for significance using the log-rank test. All data were expressed as mean± SEM. ***p < 0.001 vs. vehicle.

Supplementary Table S8. Effect of TCPOBOP on the lifespan of doxorubicin - treated mice

	Group	Number	Mean Survival ± SEM （days）	Maximum longevity (days)	Median survival time (days)	Increase （%）	*p* value
C57	Dox	9	23.300±1.606	30	24	/	/
	TCPOBOP	10	30.600±3.184	49	23	31.33	0.049*
$CAR^{-/-}$	Dox	6	23.500±2.802	34	20.5		/
	TCPOBOP	6	20.500±1.979	27	20.5		n.s.

Notes: Lifespan experiments were analyzed using Kaplan-Meier survival analysis and compared among groups, scoring for significance using the log-rank test. All data were expressed as mean± SEM. *$p < 0.001$ vs. vehicle.

Supplementary Table S9. List of primers for quantitative real-time PCR in *C. elegans*

Gene	Forward primer	Reverse primer
act-1	CGCCATCCTCCGTCTTGACTTG	GCTCAGCGGTGGTGGTGAAAG
daf-2	TGGGAGCTACGGCAGGATGATG	GCACACGGTCCGAAACGATCAC
daf-16	CGGATACCGTACTCGTGATGAT	CCAAACAGCCACCCAAATCA
sod-3	ATTAAGCGCGACTTCGGTTCCC	TCCCCAGCCAGAGCCTTGAAC
sod-2	GCTCTTCAGCCAGCTCTC	AGTATCCCAACCATCCCC
sod-1	GTGCTGTCGCTGTTCTTC	GGTCCACCATGAGTCTTTC
hsp-16.2	CTGCAGAATCTCTCCATCTGAGTC	AGATTCGAAGCAACTGCACC
daf-2	TGGGAGCTACGGCAGGATGATG	GCACACGGTCCGAAACGATCAC
daf-16	CGGATACCGTACTCGTGATGAT	CCAAACAGCCACCCAAATCA C
cat-1	CTTCTCGCCCTTCTTGCT	TCCGATGGTGATTGCTCC
clt-1	ATACTGCTGCTTCTCGTC	TCATCCCACATCTTTTTG
clt-2	AGATGTGGCGTATGTCCT	AGATGTGGCGTATGTCCT
clt-3	TCAACGGTCGCTGGAGAA	TTGCGTCACGAATGAAGAAG
mtl-1	AAGTACTGCTGTGAGGAGGC	GTTCCCTGGTGTTGATGGGT
mtl-2	CGGTTGTTAATAAATACGG	AATGTTGGAAGAGGAGCT
akt-1	AACATGGACGCAACAAGCAC	TTCCGAAGGTTCCTTGACCG
sir2.1	GCAAGAAATAACGGAGGA	TTTGAGCACGACGAAGAT
cyp-35b1	TGAACACGAGATGTGCCGAA	AACGTTTTCCGACGAGCAGA
cyp-35b2	GTTCCTCCCGCCTGTTTTCT	TTTCCTCGCATCTTGCATCC
cyp-35a2	ACTGGTGGCATTGTTTCGACTCTC	GGAATTGGTCCGACCCATAGTGTG
cyp-35C1	AAAGTGACTAACGGAGGATCTCG	CTAGCAAGAGCCGAGCTGTATTT
cyp-35a3	GCTCAACTCAGTGCTCTCCATGTC	TCCCAGGCAACTTCTCTTTCCAAC
cyp-35a4	CTGACCGTGCTTCAACTCCATACC	TCCAGCATCGACAGGGTGACC
cyp-35a5	GGGAAGGAGCCGATGGAAATCAAG	GGGAAGGAGCCGATGGAAATCAAG
gst-4	TTTGATGCTCGTGCTCTTGC	CCAAATGGAGTCGTTGGCTTC
gcs-1	AATCGATTCCTTTGGAGACC	ATGTTTGCCTCGACAATGTT
ugt-44	GCACATTTTGGTATGCTCTGCT	CGGCAACAGAAGGGTCACAT
pgp-3	GTGATGGGACTTCCTGACGG	CTTTGGGTCTCTGACAATCGC
pgp-12	CCACTCATGTACCACGGCAT	AATAGCATTCCAGCGGCAGT
pgp-13	CCGATGGCATAGACACCGAA	GCTTCTTGCACAGCCCTTTC
pgp-14	AGGAGTACGGTGCTAGCGAT	ACATCTTTGGGGCGTCATCA
cyp-14a1	CCTTTCTTGGGGTCTCATCA	AAGTAGCGGCTTGGATTGAA

Supplementary Table S10. List of primers for quantitative real-time PCR in mice

Gene	Forward primer	Reverse primer
Cyp1a2	AGTACATCTCCTTAGCCCCAG	GGGTCCGGGTGGATTCTTC
Cyp2c29	ATCTGGTCGTGTTCCTAGCG	AGTAGGCTTTGAGCCCAAATAC
Cyp2d10	GCATGGTTGTGCTAGACCTGT	GTAGGGCATACGAGCCTGG
Cyp3a11	AATTCCAAAGACAAAGTC	CCAGCAAAAATAAAGATA
Cox-3	ATGAGTCGAAGGAGTCTCTCG	GCACGGATAGTAACAACAGGGA
MRP3	CTGGGTCCCCTGCATCTAC	GCCGTCTTGAGCCTGGATAAC
Gstt1	TCTTGCTCTACCTGGCACAC	GCTCACCAAGGAAAACAGGG
Cox-2	TGCACTATGGTTACAAAAGCTGG	TCAGGAAGCTCCTTATTTCCCTT
CYP2D11	CCCAAGTGCAAGACTTCCTCG	CCAGGCATGATCTGTGTCCT
CYP2A5	TGGTCCTGTATTCACCATCTACC	ACTACGCCATAGCCTTTGAAAA
INOS	GCCAACATGCTACTGGAGGT	TGGAGCACAGCCACATTGAT
CYP2A4	CCAAAGACTTCAACCCAA	TTCTCCGAAACAATACCG
ABCC2	TTGGCACTAACCTCTGGCTG	GACCCCGATTCTCATGTCCC
Aldh3a1	CACCTGGGATCAGCCTTCAC	ACTCCACCGCTTGATGTCTC
Aldh3b3	AGAATGTCCACCAAAGGCAAAC	GTCTCCATCCAGGGCGTCAT
Ces2c	CTTGGTCATCTTAGCAGCACA	GAAGAACCCAGCATTCAGCC
CYP2A22	GGCTTTGGAGGACAACGCT	TGCCCAAGCCGAAATCCTTC
Cyp2b10	GCAAGCCATGTTGCTCCTAA	CTTGGAGCCCTGGAGATTTGG
Ugt1a1	CCTTCTGTTGTGTGTGTTCGG	CCGTCCAAGTTCCACCAAAG
Sult2a1	CCAAGGCGATCTATCTCGTG	GATCCGAATAGAACATTTCCTTTGA
Sult1a1	AGACACATCTGCCCTTGTCC	CATCCTTTGCATTTCGGGCA
Slc16a12	GGCTGAAGTGGCTGAATCCT	CCATCGGATCAAGCCAGAGT
Aldh1l2	ACGTCAAGCCCAATGACACA	TCAGCTATGAGTTGGACGGC
Slc13a5	GTCTTCCCGTGGCTAGATCC	CCAACGGGGACAGACAAACT
Slc7a8	AAGAAGCCTGACATTCCCCG	TGTGTTGCCAGTAGACACCC
Cyp7a1	CCTCCGGGCCTTCCTAAATC	CGGCTTCAAACATCACTCGG
Cyp4b1	TATCCTATGCACCAGCAGCG	TGGGGTACAGGTGGGTAGAG
Abcd2	CGTGGAAAAGAAGCCTCGGA	AGAGACTGGTCAGGGTTTGC
Cyp4a14	CCTGACTTTCTTTCGCCTGC	TGGTGGGACAAACGTCCATC
Slc2a5	ACTTCACTGTGGGGCTCATC	TACTGCTCCAAGATGGCGTG
Slc9a9	TTCTTCCAATCCGTGGGCAA	TGGTCAACAGTGCGGTAACA
Slc16a9	AAGCGCCCATTTGCTTTGAG	CCAAGAGAAACCACCACGGA

Slc43a2	GCCCAGAGGACATGGACTAC	CTGTGCATAAACGATGGGGC
Slc8a1	GGAGAGACCACCAAGACGAC	TCCCCTGCGGTGAAGTTATG
NF-κB	CCCTACGGAACTGGGCAAAT	GCGGAATCGAAATCCCCTCT
IL-1β	TCGTGCTGTCGGACCCATAT	GGTTCTCCTTGTACAAAGCTCATG
Mcp-1	AGGTCCCTGTCATGCTTC	GTGCTTGAGGTGGTTGTG
p16INK4A	GCCGTGTGCATGACGTG	TTGCCCATCATCACCTGAA
p16	GTCACACGACTGGGCGATT	ATCACCTGAATCGGGGTACG
p21	GGGTGAGGAGGAGCATGAAT	AAAGTTCCACCGTTCTCGGG
p53	CAGCCCCCTCTCTGAGTAGT	ACCCTATGAGGGCCCAAGAT

Supplementary Results

To assess the broader relevance of this study to human health, we performed senescence-associated β-galactosidase (SA-β-gal) staining on human HFF-1 cells, a well-established biomarker for cellular senescence. Microscopic analysis revealed prominent blue staining in senescent cells. A substantial number of cells exhibited the characteristic blue coloration after treatment with D-galactose and doxorubicin, indicating an increase in cellular senescence compared to the control group. However, treatment with CITCO effectively reversed this senescence-associated phenotype. These results demonstrate that the human CAR agonist can significantly alleviate D-galactose- and doxorubicin-induced cellular senescence, providing strong evidence for the anti-aging potential of CAR agonists in human cells (Supplementary Fig. S5a-d).
